# Role of Opioid-Free Anesthesia Versus Opioid-Based Anesthesia in Postoperative Pain and Opioid Consumption: A Systematic Review and Meta-Analysis

**DOI:** 10.3390/jcm15124560

**Published:** 2026-06-12

**Authors:** Akbota Ayazbekova, Abdurrehman Khan, Adina Yerzhan, Amy Monroe, Jacques E. Chelly

**Affiliations:** 1 Department of Anesthesiology and Perioperative Medicine, University of Pittsburgh, Pittsburgh, PA 15261, USA; ayazbekovaa@upmc.edu (A.A.); khana21@upmc.edu (A.K.); monroeal@upmc.edu (A.M.); 2 Department of Internal Medicine, University of Pittsburgh Medical Center Mercy Hospital, Pittsburgh, PA 15213, USA; yerzhana@upmc.edu; 3 Center for Complimentary Medicine, University of Pittsburgh, Pittsburgh, PA 15232, USA

**Keywords:** opioid-free anesthesia, balance anesthesia, opioids, dexmedetomidine, ketamine, lidocaine, postoperative pain, acute pain, chronic pain, postoperative nausea and vomiting

## Abstract

**Background/Objectives**: Opioid-free anesthesia (OFA) has emerged as a potential alternative to opioid-based anesthesia (OBA) to reduce opioid-related adverse effects. This meta-analysis compares OFA and OBA with respect to postoperative pain and opioid consumption. **Methods**: PubMed, Cochrane, and Embase libraries were searched for OFA studies published through 12 June 2025. Randomized controlled trials (RCTs) conducted on adult humans were selected; observational studies, studies including neuraxial anesthesia, and RCTs currently awaiting approval were excluded. A forest plot was used to summarize findings of a random-effects meta-analysis to compare OFA (treatment) and OBA (control). **Results**: Of 1446 citations found, twenty-nine articles met our inclusion criteria. Twenty-six studies reported pain scores with a 0–10 scale. OFA was associated with lower postoperative pain scores (Hedges’ g = –0.34; 95% CI –0.55 to –0.13; *p* < 0.001; I^2^ = 84%) but with high heterogeneity, limiting clinical significance or strong interpretation of results. Eleven trials were analyzed for opioid use, showing a small reduction with OFA (Hedges’ g = –0.55; 95% CI –1.10 to –0.005; *p* = 0.048; I^2^ = 96.22%). Subgroup outcomes favored OFA, with an overall reduction in pain found specifically in endoscopic abdominal surgeries. Some secondary outcomes also indicated potential improved recovery profiles through OFA for certain surgeries. **Conclusions**: OFA was associated with statistically significant lower postoperative pain scores, along with opioid consumption, but with a small effect size and high heterogeneity when compared to OBA. This is potentially comparable in pain control and opioid consumption with limited clinical significance. Overall, outcomes support the continued controlled study of OFA as an alternative to conventional analgesia.

## 1. Introduction

In 2000, U.S. Centers for Medicare & Medicaid Services declared pain the “fifth vital sign” and emphasized that insufficient postoperative pain control is associated with worsened perioperative outcomes [1]. Postoperative pain is a dynamic phenomenon driven by peripheral and central sensitization; when inadequately controlled, breakthrough pain can accelerate the transition to chronic postsurgical pain (CPSP) [2]. Clinically, pain impedes deep breathing and may cause coughing, discourage ambulation, and blunt participation in physiotherapy, prolonging bedrest and contributing to cardiopulmonary, thromboembolic, and infectious complications and resulting in impaired rehabilitation, prolonged length of hospital stays, and higher readmission rates [3]. Pain also drives greater postoperative opioid consumption (higher patient-controlled analgesia demands and oral morphine equivalents (OMEs), increasing opioid-related side effects such as respiratory depression, ileus, nausea/vomiting, and cardiovascular collapse [4]. High postoperative opioid consumption is also an established risk factor for the development of opioid use disorder [5]. Notably, up to 10% of previously opioid-naïve surgical patients continue to use opioids well beyond the acute period, reinforcing the need to optimize immediate postoperative analgesia and minimize avoidable exposure [6]. Consequently, pain reduction is both a patient comfort and outcome issue, necessary to optimize measurable effects on rehabilitation efficiency, prevent CPSP, and reduce opioid requirements.

Opioid-free anesthesia (OFA) has emerged as a potential alternative for improving perioperative analgesia. For the purposes of this review, OFA has been defined as an anesthetic technique in which no intraoperative opioids are given; instead, multimodal strategies combine non-opioid agents to maintain analgesia and hemodynamic stability [5]. Just as a wide variety of regimens are utilized in opioid-based anesthesia (OBA), there exists a wide variety of potential OFA strategies. Common OFA regimens include a combination of ketamine, a N-methyl-D-aspartate (NMDA) receptor antagonist, to blunt central sensitization; local and intravenous (IV) administration of anesthetics like lidocaine, bupivacaine, and ropivacaine; anti-inflammatory and non-opioid analgesics like dexmedetomidine and clonidine (α2-agonists); acetaminophen; nonsteroidal anti-inflammatory drugs (NSAIDs), and anecdotally, d-magnesium [7,8]. Studies conducted on patients undergoing laparoscopic and open abdominal, gynecologic, biliary, and colorectal procedures suggest OFA can provide effective analgesia without intraoperative opioids, measured by comparable pain scores and reduced rescue requirements [5,9,10,11]. The medications chosen for anesthesia, their doses, and their combinations differ substantially between studies; strategies are highly heterogenous and reduce the ability to make sweeping clinical recommendations.

Recent research comparing OFA and OBA has yielded mixed findings. Several randomized trials and meta-analyses report that OFA is at least as effective as OBA for acute pain management, with some demonstrating less postoperative opioid requirements and opioid-related adverse events in OFA cohorts [5,10,12,13,14]. Pooled analyses report significantly decreased morphine-equivalent consumption and pain scores in OFA patients without compromising analgesia [15]. However, other systematic reviews have found no clinically meaningful difference between OFA and conventional OBA, aside from a reduction in postoperative nausea and vomiting (PONV) with OFA [16]. These discrepancies likely reflect variations across surgical specialties, differences in OFA and OBA protocols like reliance on dexmedetomidine, ketamine, lidocaine, and regional anesthesia, limited sample size, and heterogeneity in patient risk factors [5,16]. The present study aimed to compare OFA to OBA across diverse surgical conditions with a particular focus on pain and postoperative opioid consumption, gain a better understanding of OFA’s role in anesthesia, and help define what OFA represents as a technique vis-à-vis pain management and post-operative opioid use.

## 2. Materials and Methods

This systematic review and meta-analysis were developed according to the Preferred Reporting Items for Systematic Review and Meta-Analysis (PRISMA) statement protocol (Supplementary File S1). This review was registered in the international Prospective Register of Systematic Reviews (PROSPERO) database on 9 June 2025—registration number CRD420251070516 (Supplementary File S2).

### 2.1. Eligibility Criteria

Studies that included randomized controlled trials (RCTs) conducted on adult humans were selected. These studies compared the efficacy of OFA and OBA on intraoperative and post-operative opioid consumption. Observational studies, studies that did not report an association between pain score and anesthesia type, review articles, studies including epidural and spinal anesthesia, and RCTs currently awaiting approval were excluded. Articles including epidural and spinal anesthesia were eliminated because these techniques are mostly used for anesthesia and as an alternative to intraoperative use of opioids. Articles including the use of peripheral nerve blocks were included because in most cases peripheral nerve blocks are not indicated as a part of anesthesia, but rather for postoperative pain management. Selected studies were grouped based on primary and secondary outcomes and types of surgery. Studies reporting any multimodal OFA approach were included; therefore, they were also grouped based on OFA protocols. The data were pooled using a random-effects model if at least three or more studies reported comparable outcome measures. Subgroup analyses were performed to determine whether the results and heterogeneity differed by endoscopic surgery and multimodal OFA approach type.

### 2.2. Information Sources and Search Strategy

We used PubMed, Cochrane, and Embase libraries for our systematic search strategy. The terminology used to perform the search was “Opioid AND Free AND Anesthesia”. The full search strategy, including MeSH/Emtree terms, is included in Supplementary File S3. The search was conducted on 12 June 2025. Filters were applied to limit results to English-language publications on human studies with adult patients (≥18 years old). No additional search limitations were used.

### 2.3. Selection Process

Two independent researchers manually screened trials (A.A. and A.K.). No automated tools were used for screening. Predefined studies’ full text was retrieved and reviewed in detail, and any questions and disagreements were discussed by the same two researchers. The screening procedure was based on PRISMA 2020 guidelines. Full electronic search strategies are provided in Supplementary File S3.

### 2.4. Data Collection Process

Two independent researchers manually collected data (A.A. and A.K.). No automated tools were used. Any questions and disagreements were discussed by the same two researchers, and when necessary, a third researcher (J.E.C.) made the final decision. After screening and collecting studies that met our inclusion criteria, an Excel file was created with studies grouped in separate sheets for each outcome. Information included year of publication; type of surgery; anesthetic technique for each group with dosages, highlighting the main opioid alternative in the OFA group and the main opioid used in the OBA group; postoperative analgesic regimen; pain scores; opioid consumption; PONV; separate nausea; separate vomiting; antiemetic use; rescue analgesia use; pruritus events; length of hospital stay in days; time to flatus in hours; and Quality of Recovery-40 (QoR-40) results in mean with standard deviation (SD) for each group. Finally, an additional sheet was created to assess the significance of each finding for all studies (Supplementary File S4). For studies that reported continuous outcomes as a median [interquartile range (IQR)] or median (range), the corresponding mean and SD were converted using Wan et al.’s method [17]. The primary outcomes were post-operative pain scores and mean OME. Pain was assessed using numeric rating scale and/or visual analog scale scores, with a scale of 0 = no pain to 10 = worst possible pain. Opioid consumption data were converted to OME using University of California San Francisco Opioid Equivalence Factors [18]. Although conversion methods may vary between studies, OME remains the most widely accepted standard for comparing opioid consumption across heterogeneous clinical settings. Since pain was reported at different times in each study, scores reported closest to postoperative hour 24 were used for analytical consistency, as seen in other similarly conducted meta-analyses [16]. The same 24 h postoperative period was used for opioid consumption. The secondary outcomes were nausea and vomiting reported separately; PONV; antiemetic and analgesic use; pruritus events; 24 h postoperative QoR-40; length of hospital stay in days; time to flatus in hours; and intraoperative hypertension, hypotension, bradycardia, and tachycardia. Data from individual studies were tabulated to summarize study characteristics, clinical trial periods, intervention details, and primary and secondary outcomes, opioid routes, postoperative regimens, and rescue strategies (Table 1).

### 2.5. Study Risk of Bias Assessment

Risk of bias was assessed independently by two researchers (A.A. and A.K.) using the Cochrane risk-of-bias tool for randomized trials (RoB 2). Risk of bias was rated as high concern, some concern, low concern, or no information for the following domains: D1—Bias arising from randomization process; D2—Bias due to deviations from intended intervention; D3—Bias due to missing outcome data; D4—Bias in measurement of the outcome; D5—Bias in selection of the reported results. Overall risk of bias was rated as follows: low concern only if all domains were rated low concern; some concern if at least one domain was rated some concern, while there was no high concern rating; high concern if at least one domain was rated high concern. The visualization was conducted via robvis (Supplementary File S5) [19].

The publication bias was assessed by contour-enhanced funnel plot generated by Stata version 18 software (StataCorp LLC., College Station, TX, USA). The strength of evidence for each statistically pooled outcome was assessed using the Grades of Recommendation, Assessment, Development, and Evaluation (GRADE) working group guidelines [20]. Assessment of certainty was graded using main domains of risk of bias, heterogeneity, indirectness, imprecision and publication bias. Quality of the evidence was classified as high (⊕⊕⊕⊕), moderate (⊕⊕⊕◯), low (⊕⊕◯◯), or very low (⊕◯◯◯) (Supplementary File S6).

### 2.6. Statistical Analysis

Mean differences with SD of the pain scores and OME (mg) were reported for the comparison between the OFA and OBA groups. Analyses were calculated using a random effect model because of clinical heterogeneity. Heterogeneity was assessed based on I^2^ statistic scores, τ^2^, and Cochran’s Q test. Heterogeneity was considered low if I^2^ scores were lower than 25%, medium if I^2^ scores were lower than 50%, and high if I^2^ scores were above 50%. A forest plot was built to assess the risk of bias using mean difference and Hedges’ g score, which was calculated to standardize the means and evaluate effect size, as some of the studies contained a small number of subjects. The magnitude of difference was interpreted as small when Hedges’ g was around or less than 0.2, medium when Hedges’ g was around g = 0.5, and large when Hedges’ g was around or above 0.8. A 95% confidence interval (CI) was used to report differences between groups. Subgroup analysis was performed based on pre-registered protocol to determine if OFA effect varied depending on surgery and anesthesia type. No sensitivity analysis was done. Relative risk with 95% CI was used to calculate the probability of dichotomous variables. Secondary outcomes included nausea, vomiting, PONV, antiemetic and analgesic use, length of hospital stay, pruritus events, time to flatus, QoR-40, intraoperative hypertension, hypotension, bradycardia, and tachycardia using a random-effect model. The effect measures were calculated so that negative values favored OFA, indicating lower pain and decreased OME. The meta-analysis was performed using Stata 18 software.

## 3. Results

**Figure 1:**
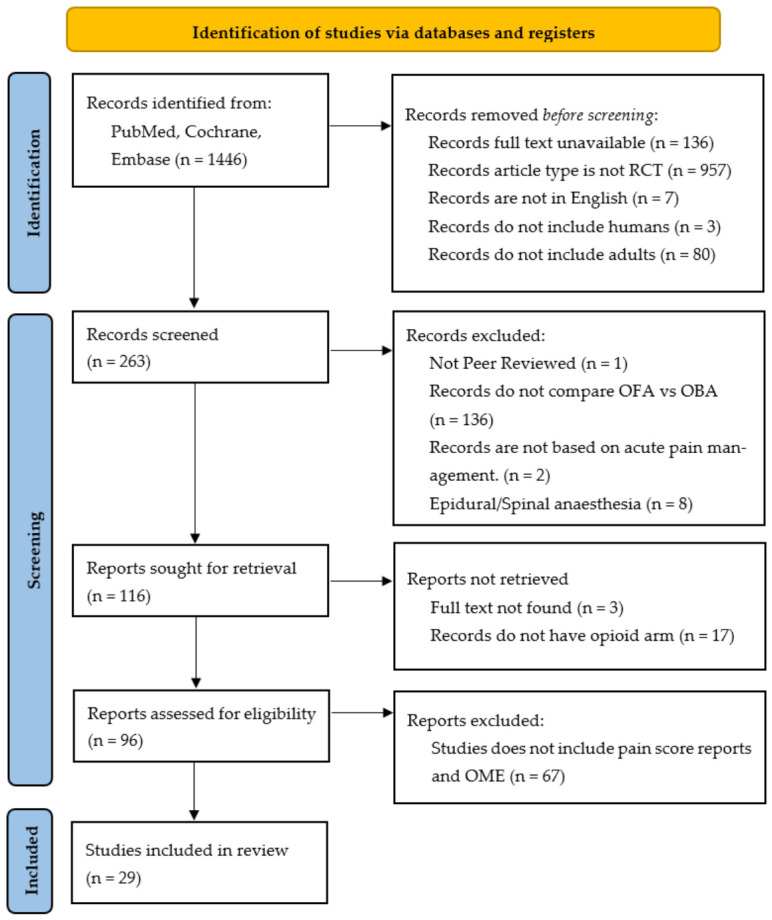
PRISMA chart showing the selection process for OFA vs. OBA articles.

Based on our literature search, 1446 citations were identified. After adding the criteria of full text available, RCTs, English-language articles, and adult human study population, 263 studies remained. The screening revealed 136 studies did not compare OFA to OBA. Furthermore, publications without full text and studies allowing the use of epidural or spinal anesthesia were excluded (Supplementary File S3). Finally, only papers that reported pain scores and OME were included. A total of 29 articles remained. The risk of bias assessment for these studies can be found in Supplementary File S5. The figure represents three articles with low concern of bias [7,21,22]. Twenty articles overall were assessed as having some concern for bias [5,8,12,13,14,23,24,25,26,27,28,29,30,31,32,33,34,35,36,37]. The other six studies were assessed as having a high risk of bias [10,11,38,39,40,41]. Included RCTs were conducted between June 2000 and September 2024. The study selection PRISMA chart is shown in Figure 1.

Overall, 29 RCTs were included in the meta-analyses [5,7,8,10,11,12,13,14,21,22,23,24,25,26,27,28,29,30,31,32,33,34,35,36,37,38,39,40,41]. Twenty-six of those reported pain scores [7,8,10,11,12,13,14,21,22,23,24,25,26,27,28,29,30,31,32,33,34,35,37,38,39,40], while 13 also reported total OME postoperatively [5,8,10,13,22,24,25,28,31,34,36,39,41]. The studies involved a total of 4119 patients, ranging from 40 to 773 per study. For each study, the nature of the effect and heterogeneity, I^2^, were established. OBA involved the use of either fentanyl, remifentanil, or sufentanil as the main opioid. OFA was based on the use either dexmedetomidine, ketamine, esketamine, or sevoflurane as an alternative to opioids. Each study’s characteristics included study period, sample size, main intraoperative medications with dosages for each group, and postoperative analgesia; overall study results are shown in Table 1.

**Table 1:** Review of selected studies.

Study	Study Period	Sample (*n*)	Intraoperative Medications (IV Unless Noted)		Postoperative Medications (IV Unless Noted)	Results
			OBA (Control)	OFA (Treatment)		
Accurso et al., 2025 [38]	October 2020–December 2022	368	Remifentanil 0.05 μg/kg/min induction → 3 to 5 ng/mL	Bilateral TAP block 20–25 mL of ropivacaine 0.3% for each side (overall 40–50 mL) Bilateral RSB 10 mL of ropivacaine 0.3% for each side (overall 20 mL) Magnesium sulphate 30 mg/kg → 8 mg/kg/h Ketorolac 90 mg (2 mL/h)	OBA: ketorolac 90 mg (2 mL/h) Morphine 0.1 mg/kg/h OFA: acetaminophen 1 g if NRS > 3	Statistically significant lower NRS score at each time point in the OFA group (*p* < 0.001). Also, HLOS, PONV, and itching events were significantly lower in OFA group.
An et al., 2022 [23]	February 2019–November 2019	101	Sufentanil 0.5 μg/kg → remifentanil 200–500 μg/h	Dexmedetomidine 0.5 µg/kg → 0.5 µg/kg/h Ketorolac 30 mg	Rescue flurbiprofen axetil	There was no statistical difference in VAS scores between the groups (*p* > 0.05); however, OFA did reduce rescue analgesic consumption after surgery (*p* < 0.05). Incidences of PONV, intestinal paralysis, pruritus, and HLOS were not significantly different between groups (*p* > 0.05).
Bae et al., 2024 [24]	Not specified	119	Remifentanil 3–5 ng/mL → 2–8 ng/mL	Dexmedetomidine 1 µg/kg over 10 min → 0.2–0.7 µg/kg/h Lidocaine 1 mg/kg → 1 mg/kg/h	Fentanyl 15 µg/kg Rescue fentanyl 50 µg or tramadol 50 mg for vNRS > 4	Opioid requirement and pain scores for 24 h after surgery was lower in the OFA group than in the OBA group (*p* = 0.036; *p* = 0.039; *p* = 0.003, respectively). PONV, LOS, and flatus events were not statistically significant.
Bakan et al., 2015 [8]	June 2012–April 2013	80	Fentanyl 2 µg/kg → remifentanil 0.25 µg/kg/min	Dexmedetomidine 0.6 µg/kg → 0.3 µg/kg/h Lidocaine 1.5 mg/kg → 2 mg/kg/h	Fentanyl 20 µg Rescue tramadol 100 mg	OFA reduced pain scores and antiemetic and analgesic use significantly (*p* = 0.028, *p* = 0.026, *p* = 0.034). However, opioid consumption, nausea, vomiting, intraoperative bradycardia, and tachycardia did not significantly change between groups.
Beloeil et al., 2021 [5]	March 2017–January 2019	314	Remifentanil 3–5 ng/mL Morphine 0.05 mg/kg	Dexmedetomidine 0.4–1.4 µg/kg/h	Morphine PCA	OFA decreased postoperative opioid consumption (*p* = 0.002), PONV (*p* = 0.01), and antiemetic use (*p* = 0.005) significantly. Intraoperative bradycardia events were significantly higher in OFA (*p* = 0.004). Other intraoperative hypotension, hypertension events, and HLOS did not differ.
Campos-Pérez et al., 2022 [12]	November 2020–March 2021	40	Fentanyl 3 µg/kg bolus → 0.003–0.006 µg/kg/min	Dexmedetomidine 1–1.5 µg/kg over 40 min → 0.3–0.7 µg/kg/min	OFA: metamizole 30 mg/kg Ketamine 0.5 mg/kg MgSO_4_ 5 mg/kg Lidocaine 1 mg/kg Paracetamol 1 g/kg every 12 h OBA: all mentioned above + buprenorphine 1 µg/kg	No significant difference in pain scores, nausea, and vomiting between groups.
Chen et al., 2023 [21]	November 2021–May 2022	77	Sufentanil 0.2–0.4 µg/kg → Remifentanil 8–10 µg/kg/h	Dexmedetomidine 0.5 µg/kg → 0.1–0.3 µg/kg/min Esketamine 0.3–0.5 mg/kg → 0.3 mg/kg/h	Flurbiprofen 2.5 mg/kg Rescue flurbiprofen 50 mg OBA: PCIA: sufentanil 2 μg/kg OFA: PCIA: Esketamine 2.5 mg/kg	OFA significantly decreased PONV events (*p* = 0.02). No difference in pain scores and analgesic use between groups.
Chen et al., 2025 [25]	March 2021–January 2022	133	Sufentanil 0.5 μg/kg → 0.025 µg/kg	Dexmedetomidine 1 μg/kg/10 min → 0.5–1.5 µg/kg/h	Flurbiprofen 2.5 mg/kg PCA bolus sufentanil 2 µg	OFA decreased pain scores significantly (*p* = 0.02). No difference in OME, PONV, and intraoperative bradycardia between groups.
Choi et al., 2022 [26]	June 2020–September 2021	75	Remifentanil 3.5 ng/mL → 0.5 ng/mL	Dexmedetomidine 0.7 µg/kg/10 min → 0.5 µg/kg/h Lidocaine 1.5 mg/kg → 1.5 mg/kg/h	PCA: fentanyl 15 µg/kg/100 mL Rescue fentanyl 0.5–1 µg/kg	Postoperative pain score, nausea, vomiting, antiemetic and analgesic use, pruritus, flatus events, and intraoperative bradycardia events were not significantly different between groups. Intraoperative hypotension events were lower (*p* = 0.039) and QoR-40 was high (*p* = 0.018) in OFA group.
Copik et al., 2024 [39]	December 2015–March 2018	50	Fentanyl 1.5 µg/kg → 1–3 µg/kg	T3–4 PVB with bupivacaine 0.3 mL/kg Lidocaine 1.5 mg/kg → 2 mg /kg/h for 2 h → 1.2 mg /kg/h to 24 h Ketamine 0.35 mg/kg → 0.2 mg /kg/h for 2 h → 0.12 mg /kg/h	Oxycodone PCA 1 mg	OFA had lower opioid consumption (*p* < 0.0001). No difference in pain, PONV, and analgesic use.
Dai et al., 2023 [40]	March 2021–April 2022	122	Sufentanil 0.5–1 µg/kg	Bilateral TAPB + QLB with 20 mL 0.20% ropivacaine per site × 4	PCIA: esketamine 50 mg + flurbiprofen 250 mg	OFA significantly reduced pain at all checkpoints (*p* < 0.001).
Feng et al., 2024 [22]	May 2022–November 2022	120	Sufentanil 0.3 µg/kg over 5 min → 0.1 µg/kg/h	Dexmedetomidine 0.6 µg/kg/10 min → 0.2–1 µg/kg/h Esketamine 0.3 mg/kg → 0.1 mg/kg	PCA sufentanil 1 µg/mL (1 µg/h basal, 2 µg bolus, 10 min lock)	Pain scores, sufentanil consumption, antiemetic use, LOS, and intraoperative HR and BP changes were similar between groups. PONV events were significantly lower in OFA (*p* = 0.031).
Hakim & Wahba, 2019 [27]	December 2017–January 2019	80	Fentanyl 1 µg/kg → 0.5 µg/kg/h	Dexmedetomidine 0.6 µg/kg / 5 min → 0.2 µg/kg/h	Rescue tramadol 0.5 mg/kg if NRS > 3	There were significantly lower pain scores (*p* = 0.02), nausea and vomiting events (*p* = 0.03), and QoR-40 (*p* = 0.03) in OFA. No significant change in intraoperative bradycardia.
Hu et al., 2024 [28]	February 2023–April 2023	72	Sufentanil 0.3 µg/kg over 5 min → 0.1 µg/kg/h	Lidocaine 1.5 mg/kg → 2 mg/kg/h Esketamine 0.15 mg/kg over 5 min → 0.10 mg/kg/h	Sufentanil 100 µg	There was no difference between groups in pain scores, postoperative opioid need, PONV, and flatus events.
Liu et al., 2023 [29]	February 2022–September 2022	66	Remifentanil 1–2 µg/kg → 0.05–0.2 µg/kg/min	Dexmedetomidine 1 µg/kg over 10 min → 0.5 µg/kg/h Esketamine 0.5 mg/kg → 0.25 mg/kg/h Lidocaine 1.5 mg/kg ICPB 7.5 mL 0.25% bupivacaine + dexmedetomidine 5 µg/kg per side	Rescue dezocine 2.5 mg (VAS 4–6) or 5 mg (>6)	There was no difference in pain scores, nausea and vomiting, pruritus, and flatus events. QoR-40 scores were statistically significantly high in OFA (*p* = 0.001).
Luo et al., 2025 [30]	September 2023–September 2024	68	Sufentanil 0.3–0.5 µg/kg → Remifentanil 6–10 µg/kg/h	Esketamine 0.3–0.5 mg/kg → 0.3–0.5 mg/kg/h	PCA: sufentanil 100 µg	OFA group had lower pain AUC (*p* = 0.001), less PONV (*p* = 0.033), and higher BP changes between groups. No difference in LOS, pruritus, and HR.
Perez et al., 2024 [31]	December 2019–June 2023	181	Fentanyl 50 µg	Dexmedetomidine 1 µg/kg → 0.4 µg/kg/h Ketamine 0.5 mg/kg Lidocaine 2 mg/kg/h	Rescue fentanyl 25–50 µg or hydromorphone 0.5 mg ward: oxycodone 5 mg every 4 h as needed (+rescue 5 mg)	No significant reduction in pain scores, OME, vomiting, antiemetic use, LOS, pruritus and bradycardia events, but OFA significantly decreased analgesic use in 24 h (*p* = 0.02).
Shirakami et al., 2006 [32]	June 2000–October 2002	51	Fentanyl 100 µg	Saline 100 µg	Flurbiprofen 50 mg Loxoprofen, po 60 mg	No significant reduction in pain scores, bradycardia, tachycardia, or hypotension events, but OFA significantly decreased analgesic use (*p* < 0.01), vomiting (*p* < 0.05), and antiemetic use (*p* < 0.05).
Swamy et al., 2025 [33]	October 2020–May 2022	70	Fentanyl 2 µg/kg	Dexmedetomidine 1 µg/kg Ketamine 25 mg	Rescue diclofenac 75 mg IV for VAS > 4	Pain score was statistically significantly reduced in OFA group (*p* < 0.0001).
Toleska & Dimitrovski, 2019 [11]	Not specified	60	Fentanyl 0.002 mg/kg	Dexamethasone 0.1 mg/kg Paracetamol 1 g Lidocaine 1 mg/kg → 2 mg/kg/h Ketamine 0.5 mg/kg Magnesium sulphate 1.5 g/h	Ketoprofen 100 mg if VAS 4–6 Tramadol 100 mg if VAS ≥ 7	Pain score was statistically significantly reduced in OFA group (*p* < 0.002).
Toleska et al., 2023 [10]	Not specified	60	Fentanyl 100 µg → 50–100 µg	Dexamethasone 0.1 mg/kg Paracetamol 1 g Lidocaine 2 mg/kg/h Ketamine 0.5 mg/kg → 0.2 mg/kg/h Magnesium sulphate 15 mg/kg/h	Thoracic epidural morphine	OFA significantly reduced pain (*p* < 0.001), opioid use (*p* = 0.0015), and PONV (*p* < 0.001). No difference in non-opioid analgesic use.
Walldén et al., 2006 [34]	April 2002–January 2003	45	Remifentanil 0.2 µg/kg/min TCI-propofol 2–4 µg/mL	Mask induction 8% sevoflurane	Rescue ketobemidone 1–2 mg for VAS > 3	Total 24 h pain, opioid consumption, and PONV events did not differ between groups.
Wang et al., 2024 [7]	May 2022–December 2022	394	Sufentanil 0.3 µg/kg → 0.1 µg/kg	Esketamine 0.3 mg/kg → 0.1 mg/kg Lidocaine 1 mg/kg Dexmedetomidine 0.5 µg/kg → 0.2 µg/kg/h	Rescue tramadol 50 mg for NRS ≥ 4	OFA decreased pain significantly (*p* = 0.017), as well as PONV (*p* < 0.001), and antiemetic use (*p* < 0.001). No difference in analgesic use and LOS.
Wang et al., 2025 [35]	July 2023–June 2024	173	Sufentanil 0.6 µg/kg	Ultrasound-guided TAP block 20 mL 0.375% ropivacaine/side dexmedetomidine 0.4 µg/kg over 10 min Esketamine 0.5 mg/kg Lidocaine 1 mg/kg	Rescue sufentanil 5 µg if VAS > 3	OFA decreased pain significantly (*p* < 0.001). No difference in PONV and analgesic use.
Xue et al., 2024 [14]	September 2021–September 2022	60	Propofol 2 mg/kg → 5–8 mg/kg/h Fentanyl 3–4 µg/kg → remifentanil 5–10 µg/kg/h	Dexmedetomidine 0.8–1 µg/kg over 10 min → 0.3–0.5 µg/kg/h Esketamine 0.3 mg/kg → 0.15 mg/kg/h	Rescue dezocine 5 mg if VAS > 3	Pain scores and antiemetic frequency were similar between groups; when counting nausea and vomiting together, OFA had fewer events (*p* = 0.039).
Yan et al., 2025 [13]	Not specified	165	Sufentanil 0.3–0.4 µg/kg → remifentanil 0.1–0.2 µg/kg/min	Dexmedetomidine 0.5 µg/kg × 15 min → 0.5 µg/kg/h Lidocaine 1.5 mg/kg → 1.5 mg/kg/h	PCA sufentanil 1 µg/h + 2 µg bolus	Pain scores, OME, and LOS were similar between groups, but OFA had fewer PONV events (*p* = 0.017).
Yu et al., 2023 [36]	Not specified	150	Remifentanil 1 µg/kg → 0.1–0.3 µg/kg/min	Dexmedetomidine 0.6 µg/kg over 10 min Lidocaine 1.5 mg/kg → 2 mg/kg/h Esketamine 0.3 mg/kg	PCIA: butorphanol 10 mg in 100 mL Rescue 2.5 mL (2.5 mg) bolus every 15 min	OFA cut rescue butorphanol requirements (*p* < 0.001), and less time to flatus in hours (*p* < 0.029) significantly. PONV events were similar between groups.
Zhou et al., 2023 [37]	May 2021–December 2021	773	Sufentanil 0.3 µg/kg → remifentanil 0.1–0.5 µg/kg/min	Dexmedetomidine 0.5 mg/kg over 10 min → 0.5 mg/kg/h Lidocaine 1.5 mg/kg → 1.5 mg/kg/h	Rescue tramadol 50–100 mg PO if NRS > 4	OFA has significantly low pain scores (*p* = 0.03), low PONV events (*p* = 0.002), and high QoC-40 scores (*p* = 0.001). There was no difference in analgesic or antiemetic use.
Ziemann-Gimmel et al., 2014 [41]	November 2011–October 2012	119	Fentanyl 0.5–1 µg/kg → intermittent fentanyl/morphine/hydromorphone boluses inhalant anesthetics	Dexmedetomidine 0.5 µg/kg → 0.1–0.3 µg/kg/h, propofol 75–150 µg/kg/min, ketamine 0.5 mg/kg bolus	Rescue hydromorphone or oxycodone PO	Post-op opioid means statistically equivalent. There was a significant decrease in PONV in OFA (*p* = 0.04).

### 3.1. Primary Outcome

#### 3.1.1. Postoperative Pain

Twenty-six studies out of 29 reported pain score associations between OFA and OBA groups [7,8,10,11,12,13,14,21,22,23,24,25,26,27,28,29,30,31,32,33,34,35,37,38,39,40]. These studies involved a total of 3526 patients, ranging from 40 to 773 per study. Supplementary File S4 shows the total sample size, number of patients in each study, and pain scores at 24 h as means with SD. Values converted from median and IQR are also indicated.

Out of 26 studies, 13 reported a statistically significant decrease in pain scores in the OFA group compared with OBA group [7,8,10,11,24,25,27,30,33,35,37,38,40]. The other 13 studies showed no difference between groups, and no study showed a reduction in pain scores in the OBA group compared with the OFA group [12,13,14,21,22,23,26,28,29,31,32,34,39]. The assessment of publication bias represents left-sided asymmetry for possible publication bias or small-study effects (Supplementary File S8). Eighteen studies’ pain reports were converted to mean with SD for analysis [7,8,12,13,14,21,22,24,25,27,29,30,32,34,35,37,38,39].

**Figure 2:**
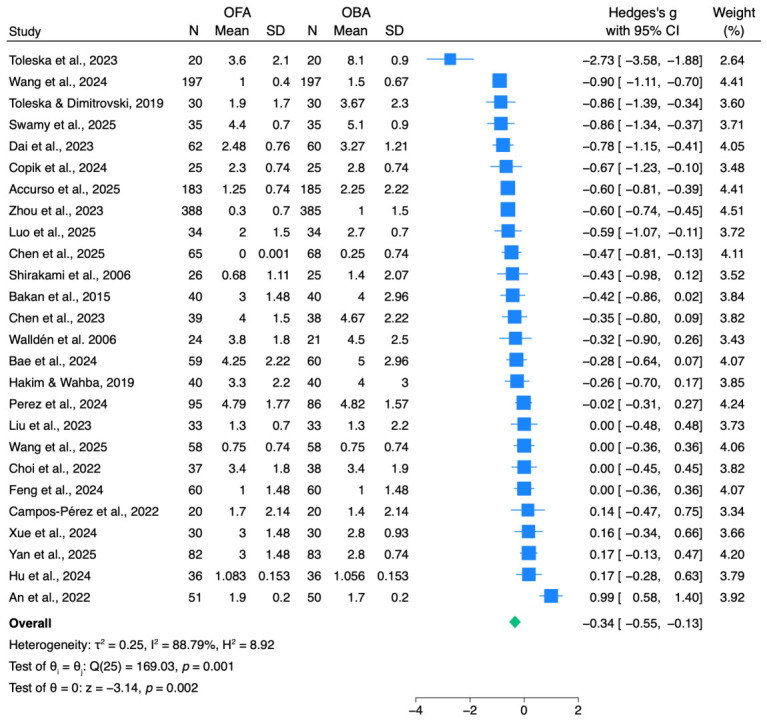
Forest plot comparing pain scores for treatment-OFA vs. control-OBA. Pooled estimates of the standardized mean difference, 95% CI are shown for the overall estimate of effect (green diamond). The standardized mean difference estimates for each study are represented as blue squares, and the blue lines represent traditional 95% CIs.

A random-effects meta-analysis found that there is a significantly small effect of reduced postoperative pain compared with OBA (Hedges’ g = –0.34; 95% CI –0.55 to –0.13; z = –3.14; *p* < 0.001). Heterogeneity was also high (I^2^ = 88.79%, *p* < 0.001). These findings suggest that while the mean pain was less in OFA, the magnitude of benefit differed across studies (Figure 2).

##### Postoperative Pain Subgroup: Endoscopic Abdominal and Pelvic Procedure

**Figure 3:**
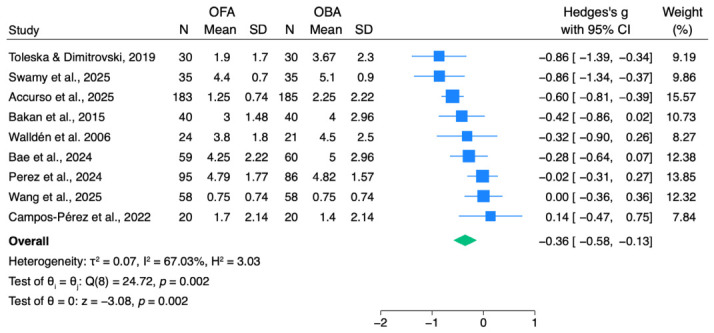
Forest plot comparing pain scores in abdominal endoscopic surgeries between treatment-OFA vs. control-OBA. Pooled estimates of the standardized mean difference, 95% CI are shown for the overall estimate of effect (green diamond). The standardized mean difference estimates for each study are represented as blue squares, and the blue lines represent traditional 95% CIs.

Fourteen out of 26 studies compared OFA versus OBA in patients undergoing abdominal [8,11,12,24,31,33,34,35,38] or pelvic endoscopic surgery [21,23,26,27,28]. Six studies reported a significant reduction in pain in the OFA group undergoing abdominal laparoscopic surgery [8,11,24,33,35,38], while the other three reported no significant difference between groups [12,31,34]. In contrast, only Hakim and Wahba [27] reported a significant reduction in pain in the OFA group undergoing pelvic laparoscopic surgery, while the other four reported no significant difference between groups (*p* = 0.64) [21,23,26,28]. Likewise, only abdominal endoscopic surgery showed significant difference between the OFA vs. OBA groups in the Restricted Maximum Likelihood (REML) model with a small-to-moderate effect size (Hedges’ g = –0.36; 95% CI –0.58 to –0.13; z = –3.08; *p* < 0.001) and there was significant heterogeneity among studies, I^2^ = 67.03%, *p* < 0.001 (Figure 3).

#### 3.1.2. OME

Thirteen studies out of 29 reported postoperative opioid consumption associations between the OFA and OBA groups [5,8,10,13,22,24,25,28,31,34,36,39,41]. Hu et al. and Wallden et al. were removed from the analysis due to the inability to convert reported postoperative values to OME [28,34]. The studies involved a total of 1469 patients, ranging from 40 to 312 per study. Supplementary File S4 shows the total sample size, number of patients in each study, and OME as mean with SD. Values converted from median and IQR are also indicated [5,13,22,24,25,36].

Out of 11 studies, five reported significantly reduced OME in the OFA group when compared with the OBA group [5,10,24,36,39]. The other eight studies showed no statistically significant difference between groups, and no study showed low OME reduction in the OBA group when compared with the OFA group [8,13,22,25,28,31,34,41]. The assessment of publication bias represents left-sided asymmetry for possible publication bias or small-study effects (Supplementary File S8).

**Figure 4:**
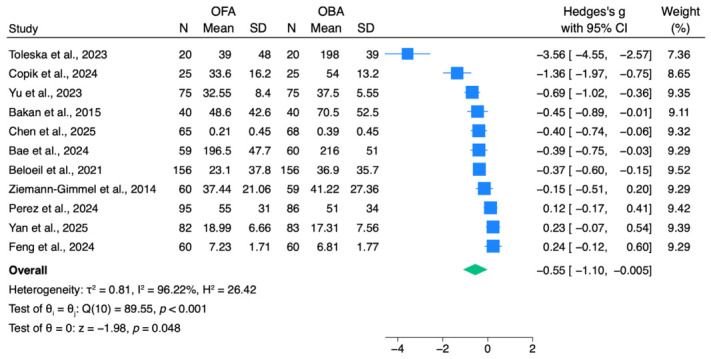
Forest plot comparing OME scores for treatment-OFA vs. control-OBA. Pooled estimates of the standardized mean difference, 95% CI are shown for the overall estimate of effect (green diamond). The mean difference estimates for each study are represented as blue squares, and the blue lines represent traditional 95% CIs.

Pooled analysis of OME between the two groups represented a statistically significant, but clinically insignificant, moderate effect size for lower opioid consumption in OFA than in OBA (Hedges’ g = –0.55; 95% CI –1.10 to –0.005; z = –1.98; *p* = 0.048). The heterogeneity score, I^2^ = 96.22%, was also very high, *p* < 0.001, due to vast variability in OBA and OFA protocols, types of surgeries, patient populations, study designs, patient comorbidities, and outcomes (Figure 4).

### 3.2. Secondary Outcome

#### 3.2.1. Postoperative Nausea and Vomiting

Eighteen studies reported PONV events, combining nausea and vomiting episodes [5,7,10,13,14,21,22,23,24,28,30,34,36,37,38,39,41], and four of them additionally reported nausea and vomiting episodes separately [14,22,34,37]. Seven other studies also reported nausea and vomiting episodes separately [8,12,26,27,29,32,35], and one study reported only vomiting [31]. Out of 18 studies that reported PONV, 11 reported significantly less episodes in OFA [5,7,10,13,14,21,22,30,37,38,41].

**Figure 5:**
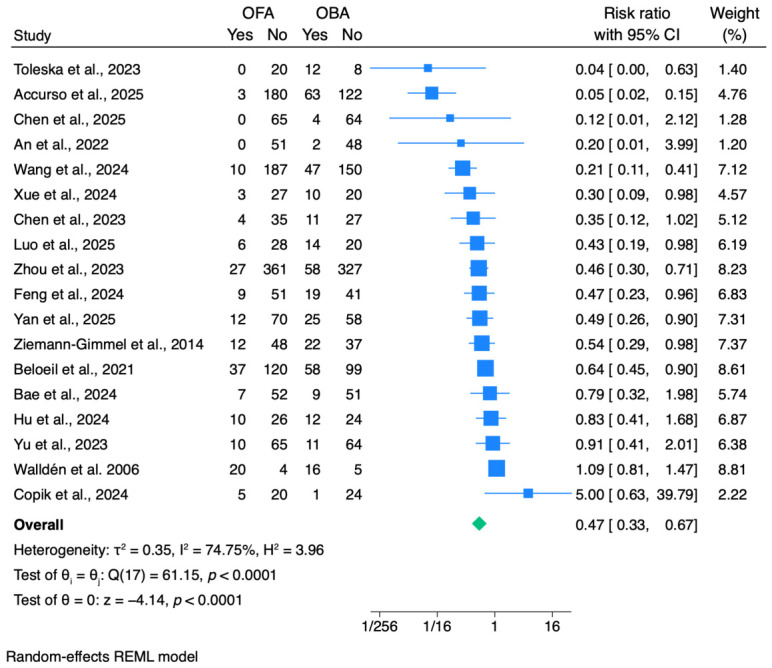
The forest plot shows pooled risk ratio for postoperative nausea and vomiting for treatment-OFA vs. control-OBA. Pooled estimates of the standardized mean difference, 95% CI are shown for the overall estimate of effect (green diamond). The standardized mean difference estimates for each study are represented as blue squares, and the blue lines represent traditional 95% CIs.

Pooled risk ratio analysis represents the risk of PONV in the OFA relative to the OBA group (risk ratio (RR) = 0.47; 95% CI 0.33 to 0.67; z = –4.14; *p* < 0.001). An RR less than one indicates higher incidence of PONV in the OBA group. Although the difference is statistically significant and the magnitude of effect is large, it is not clinically meaningful due to high heterogeneity amongst studies, I^2^ = 74.75%, *p* < 0.001 (Figure 5). Additionally, the pooled risk ratio analysis of separate nausea and vomiting events revealed a statistically significant low risk in OFA with large effect and smaller heterogeneity (Table 2, Supplementary File S7).

#### 3.2.2. Postoperative Antiemetic Use

Nine studies reported postoperative antiemetic requirements for nausea and vomiting episodes [5,7,8,14,22,26,31,32,37]. The most used antiemetic was ondansetron. Although only four studies reported a statistically significant low requirement of antiemetic in OFA [5,7,8,32], the overall pooled risk ratio showed a significant decrease in antiemetic use in OFA (RR = 0.36; 95% CI 0.2 to 0.66; z = –3.33; *p* < 0.001). The heterogeneity among studies was high, I^2^ = 75.65%, *p* < 0.001 (Table 2, Supplementary File S7).

#### 3.2.3. Rescue Non-Opioid Analgesic Use

Ten studies reported rescue non-opioid consumption, comparing OFA and OBA groups [7,8,10,21,26,31,32,35,37,39]. Different types of NSAID medications were used as non-opioid painkillers. Only three studies found a statistically significant decrease in analgesic use in the OFA group [8,31,32]. However, pooled risk ratio showed a statistically significant decrease in analgesic use in OFA overall (RR = 0.71; 95% CI 0.55 to 0.91; z = –2.65; *p* = 0.01). The heterogeneity among studies was medium, I^2^ = 46.50%, *p* = 0.03 (Table 2, Supplementary File S7).

#### 3.2.4. Other Secondary Outcomes in the Postoperative Period

Studies also compared length of hospital stay in days, pruritus episodes, time to flatus in hours, and quality of recovery assessed by the QoR-40 or QoR-15 (a shorter, 15-item version of the QoR-40) between OFA and OBA. Nine studies reported length of hospital stay; only Accurso et al. reported a statistically significant early discharge [5,7,13,22,23,24,30,31,38]. Seven studies reported time to flatus in hours; only Wang et al. and Yu et al. reported a statistically significant early time to flatus in OFA [23,24,26,28,29,35,36]. Out of six studies reporting postoperative pruritus episodes, only Accurso et al. found a statistically significant decrease in pruritus events in OFA [23,26,29,30,31,38]. Lastly, quality of recovery assessed by QoR-40 results was reported by four studies [26,27,29,37]. All studies reported significantly higher QoR-40 results in OFA. A pooled REML model showed statistically significant results favoring OFA only in overall QoR-40 results with Hedges’ g = 0.50; 95% CI 0.22 to 0.79; z = 3.46; *p* < 0.001 (Table 2, Supplementary File S7).

#### 3.2.5. Intraoperative Vital Changes

Out of thirteen studies, only one [37] study reported a significant decrease in bradycardia in OFA, and no study reported a significant difference in tachycardia [5,7,8,14,22,25,26,27,29,30,31,32]. Out of seven studies, three [7,26,30] reported significantly lower hypotensive events, and two [30,32] reported significantly higher hypertensive events in OFA intraoperatively [5,22,37]. Between pooled analysis of hemodynamic outcomes, heterogeneity varied. No significant differences between groups were observed for intraoperative hypotension (*p* = 0.13), hypertension (*p* = 0.09), bradycardia (*p* = 0.43), or tachycardia (*p* = 0.23) in pooled analysis. (Table 2, Supplementary File S7).

## 4. Discussion

The relative value of OFA and OBA in anesthesia remains the subject of ongoing debate. This meta-analysis of twenty-nine randomized trials across various surgical settings suggests with statistical significance that the use of OFA is associated with less postoperative pain scores compared to postoperative pain recorded following OBA, although this pooled estimate must be interpreted cautiously in light of very high heterogeneity (I^2^ = 88.79%) and may not have sweeping clinical significance. Across twenty-six studies analyzed, pain scores at rest were often similar following OFA and OBA (e.g., An [23]; Liu [29]; Yan [13]; Xue [14]); however, 13 studies reported that the use of OFA was associated with significantly less pain [7,8,10,11,23,24,25,27,30,33,35,37,38,40]. It could be indicated that some OFA regimens, in some surgical contexts, are associated with modest reductions in early post-operative pain, rather than OFA as a general technique to provide immediate analgesia. Subgroup analyses provide important mechanistic and practical nuances that explain these findings. OFA was associated with reduced rescue non-opioid analgesic use (RR ≈ 0.71, *p* = 0.01), suggesting that multimodal, opioid-free regimens provided sufficient baseline analgesia. This pattern reinforces the importance of α_2_-agonists and NMDA-antagonists as pharmacologically viable, synergistic components within multimodal OFA approaches, supporting the idea that the technique’s success may depend on targeting multiple pain pathways rather than simply omitting opioids. Regimen-level conclusions about specific agents should be regarded as hypothesis-generating rather than confirmatory in nature [5,6,42]. The majority of studies utilized varying medications and primarily included ketamine, esketamine, and dexmedetomidine for OFA. A small number of studies utilized sevoflurane and magnesium sulfate [34,38]. Specifically, for abdominal endoscopic surgeries, dexmedetomidine was the most prominent medication found in their OFA regimen (6/9) [8,12,24,31,33,35].

Subgroup analysis by surgical type revealed potential differences in OFA efficacy. In patients undergoing abdominal endoscopic surgery, OFA was associated with a statistically significant reduction in postoperative pain compared to OBA, with a small-to-moderate effect size (Hedges’ g = –0.36; 95% CI –0.58 to –0.13; *p* < 0.001), and a significant but moderate degree of heterogeneity across studies (I^2^ = 67.03%). In contrast, no significant difference in pain scores was found between OFA and OBA in patients undergoing pelvic endoscopic surgery, with only one study in that subgroup reporting a significant reduction in pain with OFA. Based on these findings, we may hypothesize that the analgesic benefit of OFA may be more pronounced in abdominal endoscopic procedures, and that procedure type should be considered when selecting an anesthetic strategy; although, the small number of trials and within-subgroup heterogeneity warrant additional study, and caution in extrapolating to clinical practice.

### 4.1. Postoperative Opioid Consumption

A central observation is the consistent reduction in postoperative opioid consumption, indicated by the significantly lower OME values with OFA in many trials (e.g., Beloeil [5]; Bakan [8]; Chen [25]; Copik [39]; Toleska [10]; Yu [36]). Thirteen studies reported postoperative opioid consumption values, but two were removed from analysis due to the inability to convert consumption from mg to OME for uniformity. Hu et al.’s rescue analgesia contained a mixture of opioid and non-opioid medication which was reported in combination, so no conversion factor was available. Walldén et al. utilized ketobemidone, which does not have an available conversion factor [28,34]. Eleven total studies were analyzed, and when pooled, the estimate favored OFA with a limited reduction in OME (Hedges’ g = –0.55; 95% CI –1.10 to –0.005; z = –1.98; *p* = 0.048), but with very high heterogeneity (I^2^ ~ 96.22%), underscoring the importance of regimen composition, surgical context, and co-intervention. At this level of heterogeneity, a single pooled estimate has limited interpretive value, and a uniform opioid-sparing effect of OFA across all surgical contexts is not supported by our data. As reflected by the high heterogeneity, substantial variation in surgical procedures and OFA medication regimens limited the ability to complete subgroup analyses for opioid consumption. However, the pattern of reduced opioid requirements suggests that OFA may be associated with reduced 24 h opioids requirements in some surgical contexts, such as endoscopic or other minimally invasive procedures, as suggested by our subgroup analysis indicating reduced pain, and within Enhanced Recovery After Surgery (ERAS) pathways. These observations also align with the direction of recent perioperative pain management guidelines [6,15,16].

### 4.2. Secondary Outcomes: PONV and Hemodynamics

The findings for postoperative nausea and vomiting were directionally consistent with prior systematic reviews. When analyzed as a composite PONV endpoint, the pooled estimate showed a small, statistically significant relative decrease with OFA (RR ≈ 0.47; 95% CI 0.33–0.67; *p* < 0.001); corresponding to roughly a 53% lower risk relative to OBA; however, the high heterogeneity (I^2^ ≈ 75%) limits sweeping clinical interpretation. Additionally, when nausea and vomiting events were analyzed separately, both pooled estimates favored OFA, with sizeable and statistically significant reductions in nausea (RR ≈ 0.50; 95% CI 0.36–0.71) and vomiting (RR ≈ 0.47; 95% CI 0.33–0.67) events. Similarly, the need for antiemetic rescue therapy was also reduced (RR ≈ 0.36, *p* < 0.001). The consistency in the reduction in PONV, separate nausea, vomiting, and antiemetic-use findings represents the most reproducible signal of OFA benefit identified in this review, echoing prior systematic reviews [15,16]. This is in line with the known emetogenic properties of opioids: avoiding intraoperative opioids eliminates μ-receptor activation in the chemoreceptor trigger zone, reduces gastric dysmotility, and prevents vestibular sensitization. Reductions in discrete nausea and vomiting events have tangible implications for patient experience and hospital throughput; fewer antiemetic doses translate to faster discharge readiness, especially in ambulatory and endoscopic procedures [21,26,37]. From a patient-centered perspective, this is a clinically meaningful advantage, given that PONV is among the most common, distressing postoperative complications and a cause of delayed discharge from the hospital [37].

No significant differences were observed for hypotension, hypertension, bradycardia, or tachycardia, though heterogeneity was higher among these outcomes, limiting interpretability. Overall, these findings suggest that OFA regimens did not compromise intraoperative hemodynamic stability. They do not, however, establish hemodynamic equivalence, especially in fragile patient populations. This is notable because prior critiques of OFA have suggested a risk of hemodynamic instability, particularly when dexmedetomidine is used at higher doses [42]. When OFA regimens balanced dexmedetomidine with ketamine or lidocaine, the incidence of clinically relevant hemodynamic events was comparable to OBA. This is reassuring with respect to the broad perception that OFA inherently increases hemodynamic instability but does not by itself support broader use in hemodynamically fragile patients, for whom dedicated safety trials remain needed [5,30].

From a practical point of view, both OFA and OBA practices are evolving toward a multimodal approach, which is in most cases practiced increasingly as an opioid-sparing technique. The core of the techniques being used are based on the use of α2-agonists (clonidine or dexmedetomidine), ketamine, lidocaine, acetaminophen/NSAIDs, and regional anesthesia. Dexmedetomidine inhibits adenylyl cyclase, preventing calcium from entering the nerve terminal and subsequently suppressing neural firing. This suppression leads to sedation and produces analgesic action [43]. As a full agonist of α2 adrenergic receptors with a rapid onset and short half-life, dexmedetomidine has been indicated to reduce opioid requirement in sedation regimens by up to 75% when compared to other typical sedatives such as propofol [43,44]. Using such principles, both techniques can achieve clinically important outcomes, e.g., hemodynamic stability, postoperative pain, and reduction in postoperative need for opioids. Choice of technique may also be taken into consideration, as well as the type of surgery, patient medical history, risk for perioperative hemodynamic instability, risk of PONV, respiratory risk, and frailty. However, Mercado et al., using a retrospective analysis of data from 61,249 patients undergoing non-cardiac surgery, reported that OFA was associated with an increase in acute and chronic pain and an increased risk of developing chronic pain [45]. Mercado et al. is observational and subject to confounding by indication, and the OFA cohort in that study largely did not receive non-opioid adjuncts such as ketamine, α2-agonists, lidocaine, or IV acetaminophen, and so represents a substantially different intervention from the multimodal OFA regimens evaluated in most trials included here. Rather than refuting our results, that contrast reinforces a central limitation of the present review: “OFA” as a label spans interventions that differ substantially. Across the 29 randomized trials synthesized here, the most defensible summary is that acute pain control with multimodal OFA appears generally comparable to OBA, with a modest reduction in pain in abdominal endoscopic procedures, while the magnitude of any opioid-sparing benefit is small, heterogeneous, and context-dependent.

### 4.3. Quality of Recovery

Quality of recovery outcomes, though less frequently reported, consistently favored OFA. Of the studies using QoR-40 scores, all four demonstrated significantly higher recovery quality in the OFA groups, yielding a pooled Hedges’ g ≈ 0.50 (*p* < 0.001) [21,27,37,46,47]. Because only four trials contributed to this estimate, it should be regarded as suggestive rather than definitive. Improved quality of recovery reflects multidimensional recovery with less fatigue, reduced PONV, earlier ambulation, and greater emotional well-being, all of which align with the physiological advantages of minimizing opioids. Enhanced quality of recovery is clinically consequential: patients with higher early QoR scores experience shorter stays and fewer readmissions [3,6]. This data, coupled with OFA’s opioid-sparing effects, reinforce the concept of OFA as a patient-centered strategy rather than a purely pharmacologic substitution.

### 4.4. Recent Meta-Analyses

Feenstra et al. performed a recent meta-analysis on OFA, with similar conclusions. However, our screening is more recent, including additional trials published after 2022 until June 2025, allowing for updated results. Additionally, Feenstra et al. included studies investigating and involving pediatric populations [16]. Qin et al. similarly explored pediatric trials and also did not differentiate between opioid and non-opioid rescue analgesia in their analysis [48]. Our search strategy focused on adult populations due to the differences in physiology and resultant clinical outcomes between adults and pediatric populations. Our meta-analysis contained a broader range of reported clinical outcomes with a more refined scope in population.

### 4.5. Limitations and Research Priorities

The high statistical heterogeneity (I^2^ = 98.8% for OME) decreases certainty and likely reflects protocol heterogeneity in drug combinations, doses, and timings, co-analgesic differences, and outcome timing. Underlying this, “OFA” itself is not a uniform intervention: included trials varied substantially in component drugs, doses, timing, use of regional adjuncts, and rescue criteria, and several used low-opioid rather than strictly opioid-free comparators, which means that pooled point estimates obscure underlying variation that is likely clinically important. In addition, the majority of included trials carried some or high concern for risk of bias on RoB 2 assessment, and the present analysis was designed as a conventional superiority meta-analysis; it cannot, and does not, support claims of non-inferiority or equivalence between OFA and OBA, which would require a prespecified non-inferiority framework. Two priorities emerge: standardized OFA frameworks with dose-rational protocols and clear rescue criteria, and procedure-specific RCTs with harmonized pain and OME endpoints, patient-centered recovery measures, and safety monitoring tailored to medications administered.

Contour-enhanced funnel plots for both pain and OME outcomes demonstrated leftward asymmetry, with several studies falling outside the funnel in regions of lower statistical significance, suggesting the presence of publication bias and indicating that studies favoring OFA may be overrepresented in the literature (Supplementary File S8).

Methodologically, this review spans gynecologic, gastrointestinal, colorectal, hepatobiliary, spine, and thoracic surgeries, with varied anesthetic techniques and recovery pathways. Pain scores are commonly collected at 24 h postoperatively, which allowed for a uniform timepoint at which scores were pooled for analysis. Although pain trajectories substantially differ across surgical procedures, this allowed for maximized compatibility across studies and minimized selective outcome reporting bias. Such breadth improved generalizability but amplified heterogeneity, and as indicated previously, limits generalized clinical interpretation of results. There exists wide between-study variance for both pain and OME and a real possibility of regimen-by-procedure interactions. OFA advantages may be more apparent in surgeries with pronounced visceral pain or high emetogenic risk, as suggested by the significant pain reduction observed in abdominal endoscopic procedures but not in pelvic endoscopic procedures in the present analysis. Future studies could consider procedure-specific analyses or trials to better isolate context-dependent effects. Based on high I^2^ scores, it is necessary to conduct a properly powered and large prospective study to determine the role that OFA may play as an alternative to OBA.

## 5. Conclusions

This meta-analysis suggests that OFA is a viable alternative to OBA for reducing postoperative pain and PONV, and in many cases decreasing opioid requirements. The analgesic benefit of OFA appears most consistent in abdominal endoscopic procedures, while pelvic endoscopic and other surgical contexts showed no significant difference between groups. However, variability across studies, high heterogeneity, suspected publication bias, the predominance of trials with some or high concern for risk of bias, and the substantial variation in what counts as “OFA” preclude any strong general claim that OFA is superior, equivalent, or non-inferior to OBA. A prospective, randomized OFA vs. opioid-sparing OBA trial is required to establish the role of OFA in modern anesthesia.

## Figures and Tables

**Table 2:** Pooled meta-analysis results for secondary outcomes.

Outcome	*n* of Studies	Overall	*p*	I^2^, %	z	*p*	GRADE Strength of Evidence
PONV	18	RR: 0.47 [0.33–0.67]	<0.001	74.75	−4.14	<0.001	⨁◯◯◯ Very low
Nausea events	11	RR: 0.50 [0.36–0.71]	<0.001	57.19	−3.98	<0.001	⨁⨁◯◯ Low
Vomiting events	12	RR: 0.47 [0.33–0.67]	<0.001	18.81	−4.16	0.38	⨁⨁⨁◯ Moderate
Antiemetic consumption	9	RR: 0.36 [0.20–0.66]	<0.001	75.65	−3.33	<0.001	⨁⨁◯◯ Low
Analgesic consumption	10	RR: 0.71 [0.55–0.91]	0.01	46.50	−2.65	0.03	⨁⨁◯◯ Low
Time to flatus, hours	7	Hedges’ g: −0.33 [−1.03–0.36]	0.35	95.07	−0.94	<0.001	⨁◯◯◯ Very low
Pruritus events	6	RR: 0.35 [0.11–1.15]	0.08	44.48	−1.73	0.12	⨁◯◯◯ Very low
QoR-40	4	Hedges’ g: 0.50 [0.22–0.79]	<0.001	59.52	3.46	0.05	⨁⨁◯◯ Low
Hospital length of stay, days	9	Hedges’ g: −0.21 [−0.67–0.24]	0.36	95.47	−0.91	<0.001	⨁◯◯◯ Very low
Intraoperative hypotension	7	RR: 0.73 [0.49–1.10]	0.13	76.63	−1.19	<0.001	⨁◯◯◯ Very low
Intraoperative hypertension	6	RR: 1.25 [0.97–1.60]	0.09	24.43	1.72	0.13	⨁⨁◯◯ Low
Intraoperative bradycardia	13	RR: 1.01 [0.98–1.05]	0.43	62.14	−0.71	<0.001	⨁◯◯◯ Very low
Intraoperative tachycardia	7	RR: 1.35 [0.83–2.18]	0.23	13.23	1.20	0.64	⨁⨁◯◯ Low

## Data Availability

Data is available on request.

## Supplementary Materials

The following supporting information can be downloaded at: https://www.mdpi.com/article/10.3390/jcm15124560/s1, Supplementary File S1: PRISMA 2020 checklist; Supplementary File S2: PROSPERO; Supplementary File S3: Search results; Supplementary File S4: Subgroup data; Supplementary File S5: Risk of bias; Supplementary File S6: GRADE; Supplementary File S7: Secondary outcomes; Supplementary File S8: Funnel plot.

## Abbreviations

The following abbreviations are used in this manuscript:

AUC	Area under the curve
CI	Confidence interval
CPSP	Chronic postsurgical pain
ERAS	Enhanced recovery after surgery
GRADE	Grades of recommendation, assessment, development, and evaluation
IQR	Interquartile range
IV	Intravenous
QoR-15	Quality of recovery-15 questionnaire
QoR-40	Quality of recovery-40 questionnaire
NMDA	N-methyl-D-aspartate
NSAID	Nonsteroidal anti-inflammatory drug
OBA	Opioid-based anesthesia
OFA	Opioid-free anesthesia
OME	Oral morphine equivalent
PONV	Postoperative nausea and vomiting
PRISMA	Preferred reporting items for systematic review and meta-analysis
PROSPERO	Prospective register of systematic reviews
REML	Restricted maximum likelihood
RoB 2	Cochrane risk-of-bias tool for randomized trials
RR	Relative risk
SD	Standard deviation
RCT	Randomized controlled trial
