# Supplementary material for: Role of Opioid-Free Anesthesia Versus Opioid-Based Anesthesia in Postoperative Pain and Opioid Consumption: A Systematic Review and Meta-Analysis
Source: J Clin Med. 2026 Jun 12;15(12):4560. doi: 10.3390/jcm15124560 (PMC13301896; doi:10.3390/jcm15124560)
Supplement: Supplementary file 1 [file jcm-15-04560-s001.zip › Supplementary File S2 - PROSPERO.pdf]

Role of Opioids Administered during Anesthesia in Postoperative Pain and Opioid Consumption: A Systematic Review and Meta-Analysis

Akbota Ayazbekova, Abdurrehman Khan, Adina Yerzhan, Amy Monroe, Jacques Chelly

Citation 1 change
Akbota Ayazbekova, Abdurrehman Khan, Adina Yerzhan, Amy Monroe, Jacques Chelly. Role of Opioids Administered during Anesthesia in Postoperative Pain and Opioid Consumption: A Systematic Review and Meta-Analysis. PROSPERO 2025 CRD420251070516. Available from https://www.crd.york.ac.uk/PROSPERO/view/CRD420251070516.

REVIEW TITLE AND BASIC DETAILS

Review title 1 change

Role of Opioids Administered during Anesthesia in Postoperative Pain and Opioid Consumption: A Systematic Review and Meta-Analysis

Condition or domain being studied 1 change

Postoperative Pain; Postoperative Nausea And Vomiting; Acute Pain; Opioids

Opioid-free anesthesia - evaluate the evidence for opioid based versus opioid-free anesthesia.

Rationale for the review 1 change

The rational for the review of opioid-free anesthesia is the global concern about the opioid related adverse events and dependence. Traditionally, opioid is used as one of the main components of anesthesia for intraoperative and postoperative pain management. However, there are raising concerns about opioid effects on opioid-induced hyperalgesia, development of chronic postsurgical pain and long-tern opioid dependency. , This led to increased interest in opioid-sparing and opioid-free anesthesia to reduce these mentioned issues. There are many studies that analyzed the opioid-free anesthesia; however, their results are inconclusive. The aim of our analysis is to provide with updated information about the effect of opioid-free anesthesia on postoperative pain and opioid consumption, considering increased topic popularity and publications in recent years.

1. Angst, M. S., & Clark, J. D. (2006). Opioid-induced hyperalgesia: a qualitative systematic review. Anesthesiology, 104(3), 570–587. https://doi.org/10.1097/00000542-200603000-00025

2. Hug C. C., Jr (1992). Opioids: clinical use as anesthetic agents. Journal of pain and symptom management, 7(6), 350–355. https://doi.org/10.1016/0885-3924(92)90088-y

Review objectives 1 change

The aim of this review is to study the updated research on opiate-free anesthesia compared to opiate-based anesthesia and investigate the differences and efficacy on postoperative acute pain and opioid consumption.

Keywords 1 change

Opioid-free anesthesia; Balance anesthesia; Opioids; Dexmedetomidine; Ketamine; Lidocaine; Postoperative pain; Acute pain; Postoperative nausea and vomiting; Complications

Country

United States of America

ELIGIBILITY CRITERIA

Population

Included
Adults over 18 years of age

Excluded
Children, adolescents younger 18 years of age

Intervention(s) or exposure(s)

Included
Participants undergoing surgery with a non-opioid alternative anesthesia.

Comparator(s) or control(s) 1 change

Included
Participants undergoing surgery with a standard opioid-based anesthesia

Study design

Only randomized study types will be included.

Included
Randomized-controlled studies

Excluded
Observation studies

Randomized-controlled studies pending approval

Spinal or epidural anesthesia

Context

Studies reporting postoperative pain and opioid consumption reports after the surgery

TIMELINE OF THE REVIEW

Date of first submission to PROSPERO
09 June 2025

Review timeline 1 change
Start date: 9 June 2025. End date: 31 October 2025.

Date of registration in PROSPERO
09 June 2025

AVAILABILITY OF FULL PROTOCOL

Availability of full protocol 1 change
A full protocol has been written and uploaded to PROSPERO. The protocol may be accessed through this link https://www.crd.york.ac.uk/PROSPEROFILES/69e7d08ae949ea27e5976d5eec937f7a.pdf.

SEARCHING AND SCREENING

Search for unpublished studies
Only published studies will be sought.

Main bibliographic databases that will be searched 1 change
The main databases to be searched are *CENTRAL - Cochrane Central Register of Controlled Trials*, *Embase.com*, *MEDLINE* and *PubMed*.

Search language restrictions
The review will only include studies published in English.

Search date restrictions
There are no search date restrictions.

Other methods of identifying studies 1 change
Other studies will be identified by: *looking through all the articles that cite the papers included in the review ("snowballing" or forward citation searching)*, *reference list checking*, *reference list checking (backward citation searching)* and *searching trial or study registers*.

Link to search strategy
A full search strategy has been uploaded to PROSPERO. The PDF may be accessed through this link https://www.crd.york.ac.uk/PROSPEROFILES/7f9cc0ca1495e7188a37b65a0bf2e3e9.pdf.

Selection process
Studies will be screened by one person (or a machine) and checked by at least one other person (or machine).

Other relevant information about searching and screening

None

DATA COLLECTION PROCESS

Data extraction from published articles and reports 1 change

Data will be extracted by one person (or a machine) and checked by at least one other person (or machine).

Authors will be asked to provide any required data not available in published reports.

Study risk of bias or quality assessment 1 change

Risk of bias will be assessed using: *Cochrane RoB-2*

Data will be assessed by one person (or a machine) and checked by at least one other person (or machine).

Additional information will be sought from study investigators if required information is unclear or unavailable in the study publications/reports.

Reporting bias assessment

Risk of bias due to missing results will be assessed

Certainty assessment

Grading of Recommendations Assessment, Development and Evaluation (GRADE) approach will be used to assess certainty.

OUTCOMES TO BE ANALYSED

Main outcomes

Main outcome is to investigate the differences and efficacy on postoperative pain and opioid consumption between opiate-free anesthesia and opiate-based anesthesia.

Additional outcomes 1 change

Nausea and vomiting reported separately, PONV, antiemetic and analgesic use, pruritus events, and QoR-40 24-hour postoperatively, length of hospital stay in days, time to flatus in hours, intraoperative hypertension, hypotension, bradycardia, and tachycardia.

PLANNED DATA SYNTHESIS

Strategy for data synthesis

Random-effects model to account for clinical and methodological heterogeneity, will be conducted in STATA software. Sub-group and sensitivity analysis will be performed to find additional to find additional moderators that might explain variations in effect sizes.

CURRENT REVIEW STAGE

Stage of the review at this submission 1 change

Review stage	Started	Completed
Pilot work	✓	✓
Formal searching/study identification	✓	✓
Screening search results against inclusion criteria	✓	✓
Data extraction or receipt of IPD	✓	✓
Risk of bias/quality assessment	✓	✓
Data synthesis	✓	✓

Review status
The review is completed.

Publication of review results 1 change
Results of the review will be published in English.

Journal publication
Not yet published in a journal but will be in future.

REVIEW AFFILIATION, FUNDING AND PEER REVIEW

Review team members 1 change
Dr Akbota Ayazbekova (review guarantor and contact) ORCID: 0009-0007-4351-5986. Department of Anesthesiology and Perioperative Medicine. United States of America.

No conflict of interest declared.

Abdurrehman Khan. ORCID: 0009-0001-0698-9759. Department of Anesthesiology and Perioperative Medicine. United States of America.

No conflict of interest declared.

Dr Adina Yerzhan. University of Pittsburgh Medical Center Mercy Hospital. United States of America.

No conflict of interest declared.

Amy Monroe. Department of Anesthesiology and Perioperative Medicine. United States of America.

No conflict of interest declared.

Professor Jacques Chelly. ORCID: 0000-0001-5715-5482. Director of Acute Pain Research, Departments of Anesthesiology and Orthopaedic Surgery, Co-Director of the Center for Innovation in Pain care, Director of the Center for Complementary and Alternative Medicine, University of Pittsburgh, School of Medicine, University of Pittsburgh Medical Center. United States of America.

No conflict of interest declared.

Named contact

Dr Akbota Ayazbekova (ayazbekovaa@upmc.edu). ORCID: 0009-0007-4351-5986. Department of Anesthesiology and Perioperative Medicine. United States of America.

Review affiliation

University of Pittsburgh, Department of Anesthesiology and Perioperative Medicine, Pittsburgh, PA, USA

Funding source

Review has no funding and no agreed support from an academic institution and is done in authors' own time.

Peer review

The protocol will be reviewed internally by the study team, including clinical and methodological experts in anesthesiology and pain management.

ADDITIONAL INFORMATION

Review conflict of interest

Declared individual interests are recorded under team member details.. No additional interests are recorded for this review.

Medical Subject Headings 1 change

Anesthesia; Analgesics, Opioid; Adult; Humans

Revision note 1 change

These changes were made to incorporate the latest progress and ensure the document reflects the current state of the work.

SIMILAR REVIEWS

Check for similar records already in PROSPERO

PROSPERO identified a number of existing PROSPERO records that were similar to this one (last check made on 9 June 2025). These are shown below along with the reasons given by that the review team for the reviews being different and/or proceeding.

- Opioid free anesthesia in thoracic surgery: a systematic review and meta-analysis [published 6 August 2022] [CRD42022344504]. The review was acknowledged as **similar** but the authors opted to continue because *the review looks at additional or different outcomes*, *the review will be more up to date*
- Opioid-free anesthesia versus opioid-based anesthesia in patients undergoing cardiothoracic surgery: a meta-analysis & systematic review [published 31 July 2022] [CRD42022348196]. The review was acknowledged as **similar** but the authors opted to continue because *there are differences in population*, *the review looks at additional or different outcomes*, *the review will be more up to date*

- Opioid-free anesthesia versus opioid-based anesthesia for laparoscopic cholecystectomy: a sistematic review and meta-analysis [published 22 July 2024] [CRD42024568652]. The review was acknowledged as **similar** but the authors opted to continue because *there are differences in population*, *the review looks at additional or different outcomes*, *the review will be more up to date*

PROSPERO version history 1 change

- Version 2.0, published 19 Nov 2025
- Version 1.0, published 09 Jun 2025

Disclaimer

The content of this record displays the information provided by the review team. PROSPERO does not peer review registration records or endorse their content.

PROSPERO accepts and posts the information provided in good faith; responsibility for record content rests with the review team. The guarantor for this record has affirmed that the information provided is truthful and that they understand that deliberate provision of inaccurate information may be construed as scientific misconduct.

PROSPERO does not accept any liability for the content provided in this record or for its use. Readers use the information provided in this record at their own risk.

Any enquiries about the record should be referred to the named review contact
