# Supplementary material for: Role of Opioid-Free Anesthesia Versus Opioid-Based Anesthesia in Postoperative Pain and Opioid Consumption: A Systematic Review and Meta-Analysis
Source: J Clin Med. 2026 Jun 12;15(12):4560. doi: 10.3390/jcm15124560 (PMC13301896; doi:10.3390/jcm15124560)
Supplement: Supplementary file 1 [file jcm-15-04560-s001.zip › Supplementary File S5 - Risk of bias.pdf]

Supplementary File S5 - **Risk of bias**

Study	Risk of bias domains: D1	D2	D3	D4	D5	Overall
Accurso et al., 2025	+	-	+	X	-	X
An et al., 2022	-	+	+	+	+	-
Bae et al., 2024	-	+	+	+	+	-
Bakan et al., 2015	-	+	+	+	+	-
Beloeil et al., 2021	+	-	+	+	+	-
Campos-Pérez et al., 2022	-	-	+	+	-	-
Chen et al., 2023	+	+	+	+	+	+
Chen et al., 2025	-	+	+	+	+	-
Choi et al., 2022	-	+	+	+	+	-
Copik et al., 2024	-	X	-	-	+	X
Dai et al., 2023	-	X	+	-	+	X
Feng et al., 2024	+	+	+	+	+	+
Hakim & Wahba, 2019	+	+	+	+	-	-
Hu et al., 2024	-	+	+	+	+	-
Liu et al., 2023	-	+	-	+	+	-
Luo et al., 2025	+	-	+	+	+	-
Perez et al., 2024	-	+	-	+	+	-
Shirakami et al., 2006	-	+	+	+	-	-
Swamy et al., 2025	-	+	+	+	-	-
Toleska & Dimitrovski, 2019	X	-	+	-	-	X
Toleska et al., 2023	X	-	+	X	-	X
Walldén et al. 2006	-	-	-	+	-	-
Wang et al., 2024	+	+	+	+	+	+
Wang et al., 2025	-	+	+	+	-	-
Xue et al., 2024	-	-	+	-	+	-
Yan et al., 2025	-	+	+	+	+	-
Yu et al., 2023	-	+	+	+	+	-
Zhou et al., 2023	-	+	+	+	+	-
Ziemann-Gimmel et al., 2014	-	X	-	+	+	X

Domains:
D1: Bias arising from the randomization process.
D2: Bias due to deviations from intended intervention.
D3: Bias due to missing outcome data.
D4: Bias in measurement of the outcome.
D5: Bias in selection of the reported result.

Judgement
X High
- Some concerns
+ Low
