# Supplementary material for: Role of Opioid-Free Anesthesia Versus Opioid-Based Anesthesia in Postoperative Pain and Opioid Consumption: A Systematic Review and Meta-Analysis
Source: J Clin Med. 2026 Jun 12;15(12):4560. doi: 10.3390/jcm15124560 (PMC13301896; doi:10.3390/jcm15124560)
Supplement: Supplementary file 1 [file jcm-15-04560-s001.zip › Supplementary File S6 - GRADE.pdf]

Author(s): Akbota Ayazbekova, Abdurrehman Khan, Adina Yerzhan, Amy Monroe, Jacques E Chelly
Question: Opioid-free anesthesia compared to Opioid-based anesthesia for diverse surgeries to reduce pain and postoperative opioid consumption
Setting: operating room
Bibliography:

Certainty assessment							№ of patients		Effect		Certainty	Importance
**№ of studies**	**Study design**	**Risk of bias**	**Inconsistency**	**Indirectness**	**Imprecision**	**Other considerations**	**Opioid-free anesthesia**	**Opioid-based anesthesia**	**Relative (95% CI)**	**Absolute (95% CI)**		
**Pain (assessed with: points)**												
26	randomised trials	serious[a]	very serious[b]	not serious	not serious	publication bias strongly suspected[c]	1769	1757	-	SMD **0.34 SD lower** (0.55 lower to 0.13 lower)	⊕◯◯◯ Very low[a,b,c]	CRITICAL
**OME (assessed with: mg)**												
11	randomised trials	serious[a]	very serious[b]	not serious	not serious	publication bias strongly suspected[c]	737	732	-	SMD **0.55 SD lower** (1.1 lower to 0.005 lower)	⊕◯◯◯ Very low[a,b,c]	CRITICAL
**PONV (assessed with: events)**												
18	randomised trials	serious[a]	serious[b]	not serious	not serious	publication bias strongly suspected[c]	175/1585 (11.0%)	394/1583 (24.9%)	**RR 0.47** (0.33 to 0.67)	**132 fewer per 1,000** (from 167 fewer to 82 fewer)	⊕◯◯◯ Very low[a,b,c]	IMPORTANT
**Nausea (assessed with: events)**												
11	randomised trials	not serious	serious[b]	not serious	not serious[d]	publication bias strongly suspected[c]	94/756 (12.4%)	172/749 (23.0%)	**RR 0.50** (0.36 to 0.71)	**115 fewer per 1,000** (from 147 fewer to 67 fewer)	⊕⊕◯◯ Low[b,c,d]	IMPORTANT
**Vomiting (assessed with: events)**												
12	randomised trials	not serious	serious[e]	not serious	not serious	publication bias strongly suspected strong association[c]	58/851 (6.8%)	114/835 (13.7%)	**RR 0.47** (0.33 to 0.67)	**72 fewer per 1,000** (from 91 fewer to 45 fewer)	⊕⊕⊕◯ Moderate[c,e]	IMPORTANT
**Antiemetic use (assessed with: events)**												
9	randomised trials	not serious	very serious[b]	not serious	not serious	publication bias strongly suspected strong association[c]	107/1018 (10.5%)	186/1008 (18.5%)	**RR 0.36** (0.20 to 0.66)	**118 fewer per 1,000** (from 148 fewer to 63 fewer)	⊕⊕◯◯ Low[b,c]	IMPORTANT
**Time to flatus (assessed with: hour)**												
7	randomised trials	serious[a]	very serious[b]	not serious	serious[d]	publication bias strongly suspected[c]	349	349	-	SMD **0.33 SD lower** (1.03 lower to 0.36 higher)	⊕◯◯◯ Very low[a,b,c,d]	IMPORTANT
**Pruritus (assessed with: events)**												
6	randomised trials	serious[a]	not serious	not serious	serious[d]	publication bias strongly suspected[c]	12/424 (2.8%)	65/420 (15.5%)	**RR 0.35** (0.11 to 1.15)	**101 fewer per 1,000** (from 138 fewer to 23 more)	⊕◯◯◯ Very low[a,c,d]	IMPORTANT
**Quality of recovery (assessed with: points; Scale from: 40 to 200)**												
4	randomised trials	serious[a]	serious[b]	not serious	not serious	none	498	496	-	SMD **0.5 SD higher** (0.22 higher to 0.79 higher)	⊕⊕◯◯ Low[a,b]	IMPORTANT
**Hospital length of stay (assessed with: days)**												
9	randomised trials	serious[a]	very serious[b]	not serious	serious[d]	publication bias strongly suspected[c]	918	912	-	SMD **0.21 SD lower** (0.67 lower to 0.24 higher)	⊕◯◯◯ Very low[a,b,c,d]	IMPORTANT

**Hypotension (assessed with: mmHg)**												
7	randomised trials	not serious	very serious[b]	not serious	serious[d]	publication bias strongly suspected[c]	185/899 (20.6%)	216/896 (24.1%)	**RR 0.73** (0.49 to 1.10)	**65 fewer per 1,000** (from 123 fewer to 24 more)	⊕◯◯◯ Very low[b,c,d]	IMPORTANT
**Hypertension (assessed with: mmHg)**												
6	randomised trials	not serious	not serious	not serious	serious[d]	publication bias strongly suspected[c]	210/862 (24.4%)	168/858 (19.6%)	**RR 1.25** (0.97 to 1.60)	**49 more per 1,000** (from 6 fewer to 117 more)	⊕⊕◯◯ Low[c,d]	IMPORTANT
**Bradycardia (assessed with: beats/min)**												
15	randomised trials	serious[a]	serious[b]	not serious	serious[d]	publication bias strongly suspected[c]	107/1320 (8.1%)	140/1310 (10.7%)	**RR 0.84** (0.51 to 1.37)	**17 fewer per 1,000** (from 52 fewer to 40 more)	⊕◯◯◯ Very low[a,b,c,d]	IMPORTANT
**Tachycardia (assessed with: beats/min)**												
7	randomised trials	not serious	not serious	not serious	serious[d]	publication bias strongly suspected[c]	59/778 (7.6%)	47/774 (6.1%)	**RR 1.35** (0.83 to 2.18)	**21 more per 1,000** (from 10 fewer to 72 more)	⊕⊕◯◯ Low[c,d]	IMPORTANT
**Analgesics use (assessed with: events)**												
10	randomised trials	serious[a]	not serious	not serious	not serious	publication bias strongly suspected[c]	138/927 (14.9%)	196/911 (21.5%)	**RR 0.71** (0.55 to 0.91)	**62 fewer per 1,000** (from 97 fewer to 19 fewer)	⊕⊕◯◯ Low[a,c]	CRITICAL
**Abdominal laparoscopic surgeries (assessed with: points)**												
9	randomised trials	serious[a]	serious[b]	not serious	not serious	publication bias strongly suspected[c]	544	535	-	SMD **0.36 SD lower** (0.58 lower to 0.13 lower)	⊕◯◯◯ Very low[a,b,c]	CRITICAL
**Pelvic laparoscopic surgeries (assessed with: points)**												
5	randomised trials	serious[a]	very serious[b]	not serious	serious[d]	none	203	202	-	SMD **0.11 SD higher** (0.36 lower to 0.59 higher)	⊕◯◯◯ Very low[a,b,d]	IMPORTANT

CI: confidence interval; **RR:** risk ratio; **SMD:** standardised mean difference

Explanations

a. Some OFA studies lack blinding or clear randomization.
b. Large heterogeneity
c. Asymmetry of funnel plot.
d. Wide CI
e. Possible heterogeneity due to protocol variation
