# Supplementary material for: Role of Opioid-Free Anesthesia Versus Opioid-Based Anesthesia in Postoperative Pain and Opioid Consumption: A Systematic Review and Meta-Analysis
Source: J Clin Med. 2026 Jun 12;15(12):4560. doi: 10.3390/jcm15124560 (PMC13301896; doi:10.3390/jcm15124560)
Supplement: Supplementary file 1 [file jcm-15-04560-s001.zip › Supplementary File S7 - Secondary outcomes.pdf]

Supplementary File S7 - Secondary outcomes

Study	OFA Yes	OFA No	OBA Yes	OBA No	Risk ratio with 95% CI	Weight (%)
Toleska et al., 2023	0	20	12	8	0.04 [0.00, 0.63]	1.40
Accurso et al., 2025	3	180	63	122	0.05 [0.02, 0.15]	4.76
Chen et al., 2025	0	65	4	64	0.12 [0.01, 2.12]	1.28
An et al., 2022	0	51	2	48	0.20 [0.01, 3.99]	1.20
Wang et al., 2024	10	187	47	150	0.21 [0.11, 0.41]	7.12
Xue et al., 2024	3	27	10	20	0.30 [0.09, 0.98]	4.57
Chen et al., 2023	4	35	11	27	0.35 [0.12, 1.02]	5.12
Luo et al., 2025	6	28	14	20	0.43 [0.19, 0.98]	6.19
Zhou et al., 2023	27	361	58	327	0.46 [0.30, 0.71]	8.23
Feng et al., 2024	9	51	19	41	0.47 [0.23, 0.96]	6.83
Yan et al., 2025	12	70	25	58	0.49 [0.26, 0.90]	7.31
Ziemann-Gimmel et al., 2014	12	48	22	37	0.54 [0.29, 0.98]	7.37
Beloeil et al., 2021	37	120	58	99	0.64 [0.45, 0.90]	8.61
Bae et al., 2024	7	52	9	51	0.79 [0.32, 1.98]	5.74
Hu et al., 2024	10	26	12	24	0.83 [0.41, 1.68]	6.87
Yu et al., 2023	10	65	11	64	0.91 [0.41, 2.01]	6.38
Walldén et al. 2006	20	4	16	5	1.09 [0.81, 1.47]	8.81
Copik et al., 2024	5	20	1	24	5.00 [0.63, 39.79]	2.22
**Overall**					0.47 [0.33, 0.67]	

Heterogeneity: $\tau^2 = 0.35$, $I^2 = 74.75\%$, $H^2 = 3.96$

Test of $\theta_i = \theta_j$: $Q(17) = 61.15$, $p < 0.0001$

Test of $\theta = 0$: $z = -4.14$, $p < 0.0001$

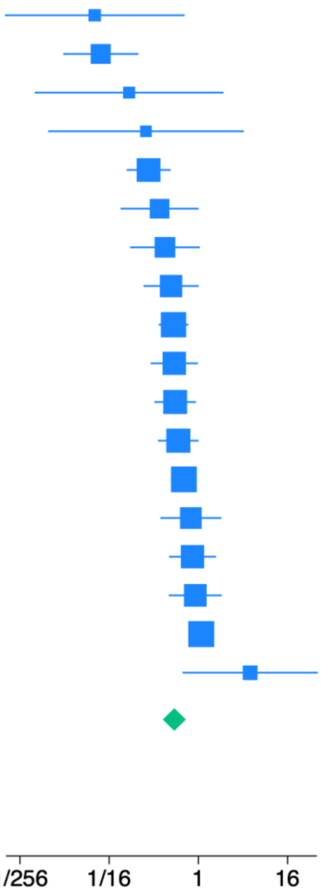

Random-effects REML model

a) Forest plot showing pooled risk ratio for **PONV** for treatment-OFA vs. control-OBA.

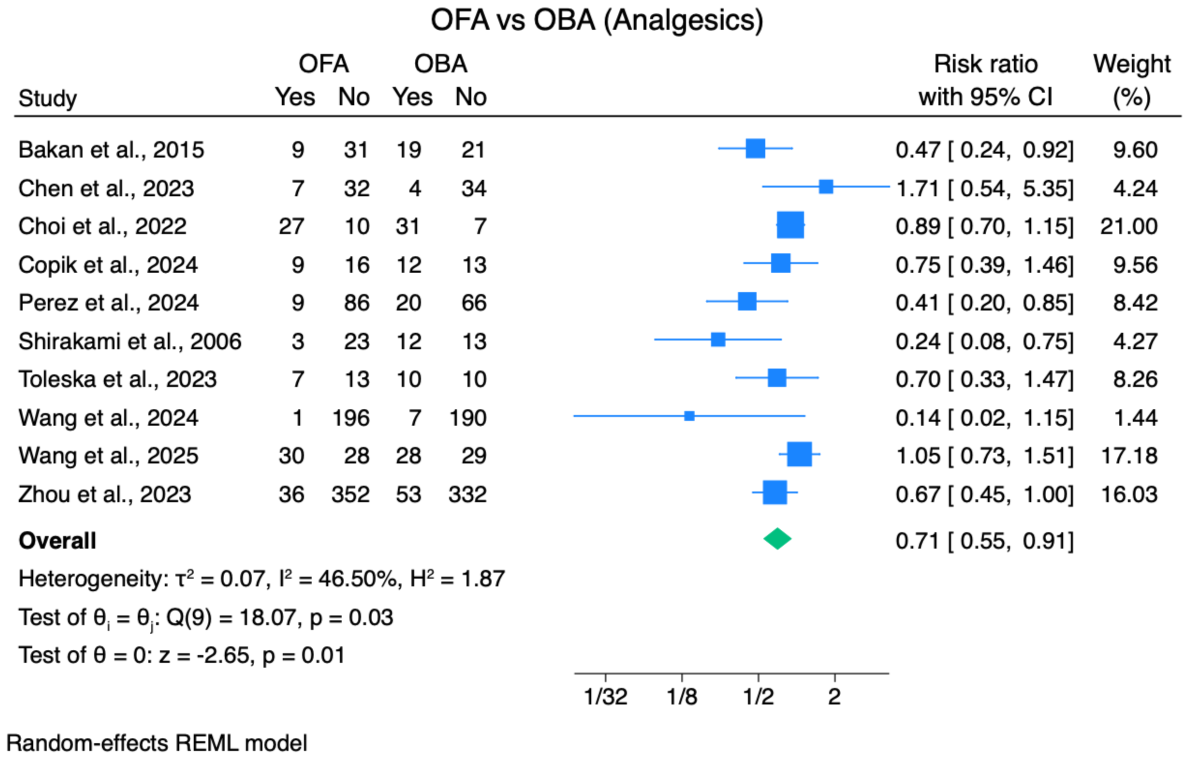

b) Forest plot showing pooled risk ratio for **non-opioid analgesic** use for treatment-OFA vs. control-OBA.

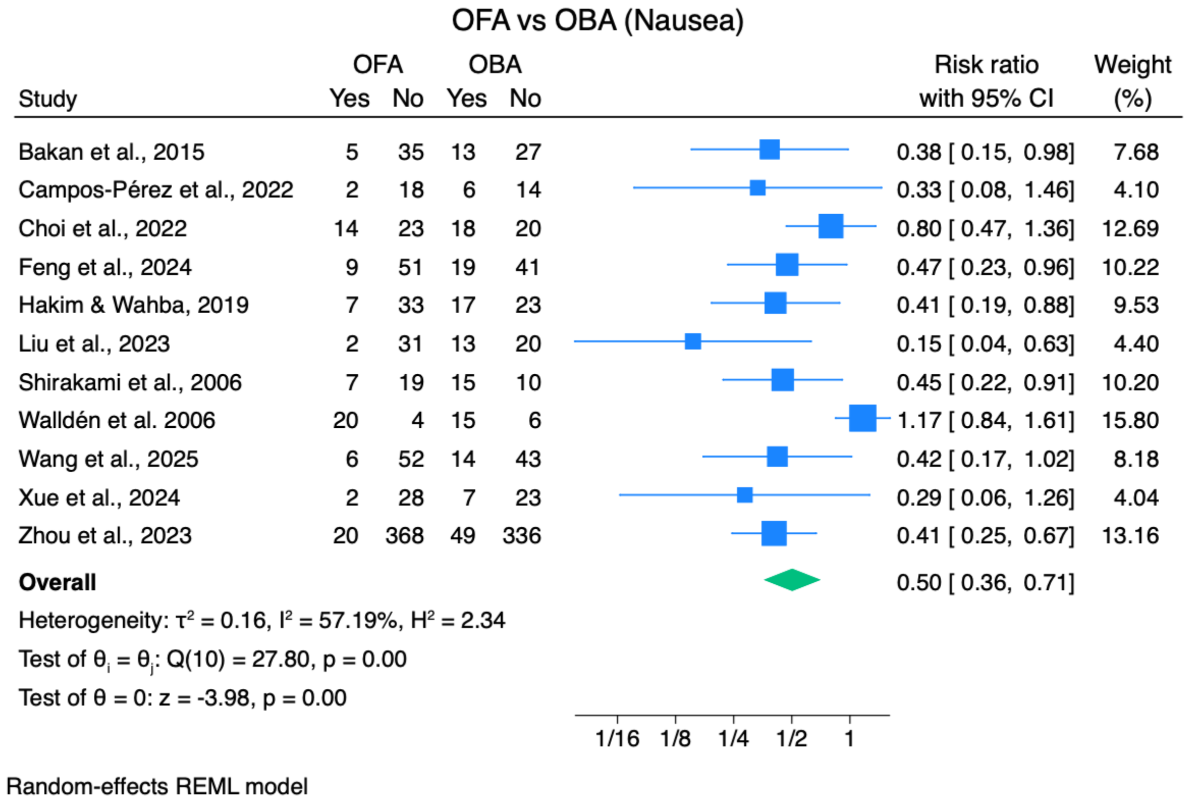

c) Forest plot showing pooled risk ratio for **postoperative nausea** for treatment-OFA vs. control-OBA.

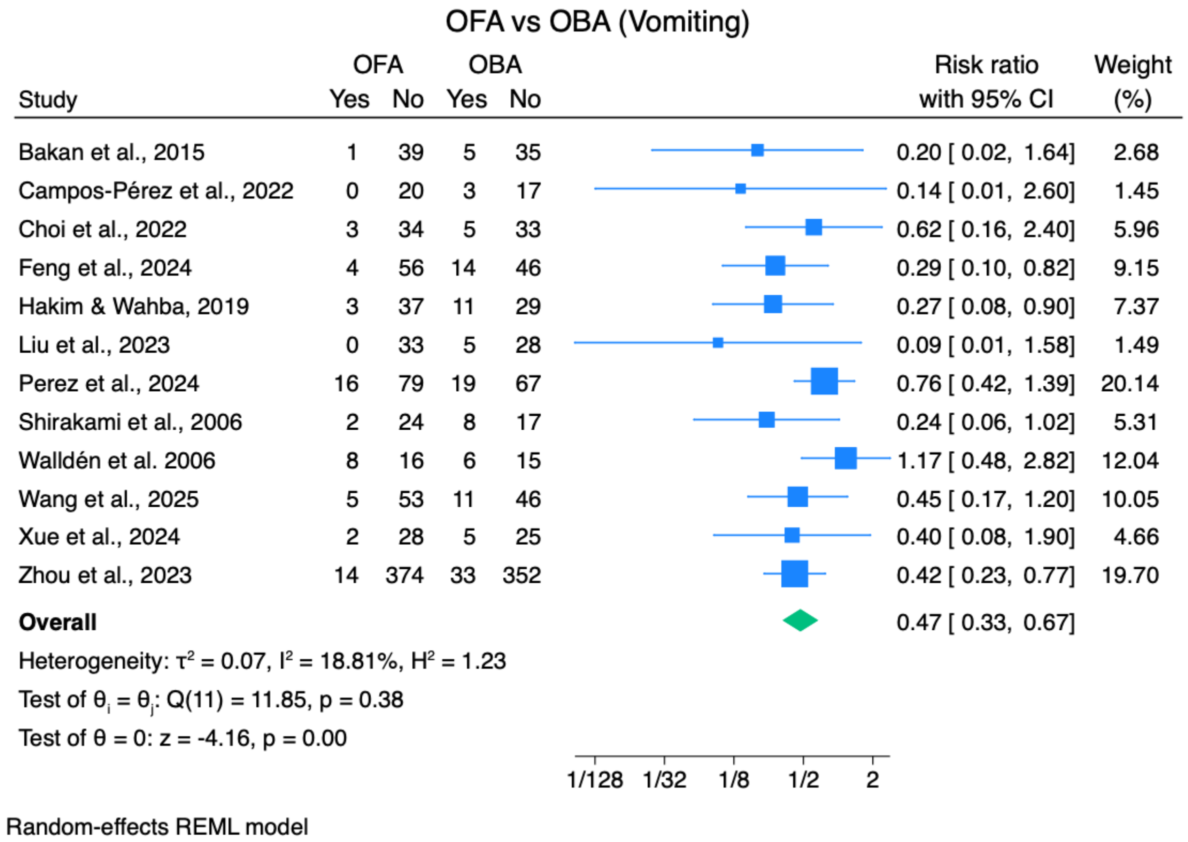

d) Forest plot showing pooled risk ratio for **postoperative vomiting** for treatment-OFA vs. control-OBA.

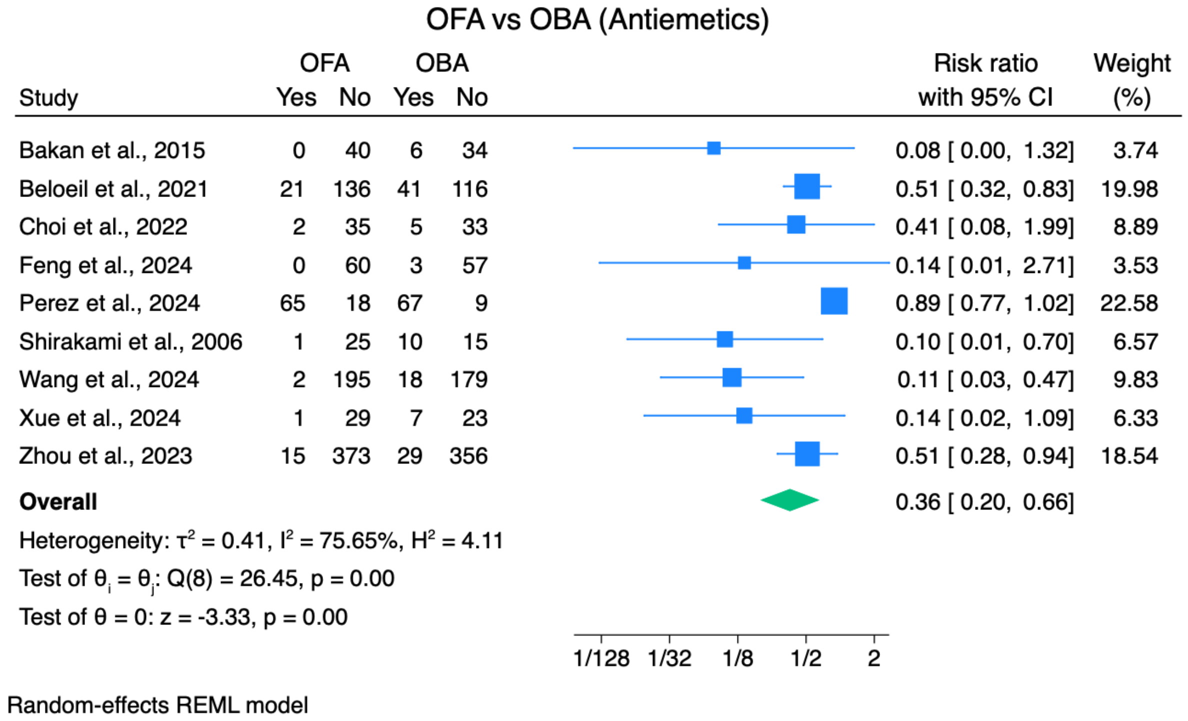

e) Forest plot showing pooled risk ratio for **postoperative antiemetic use** for treatment-OFA vs. control-OBA.

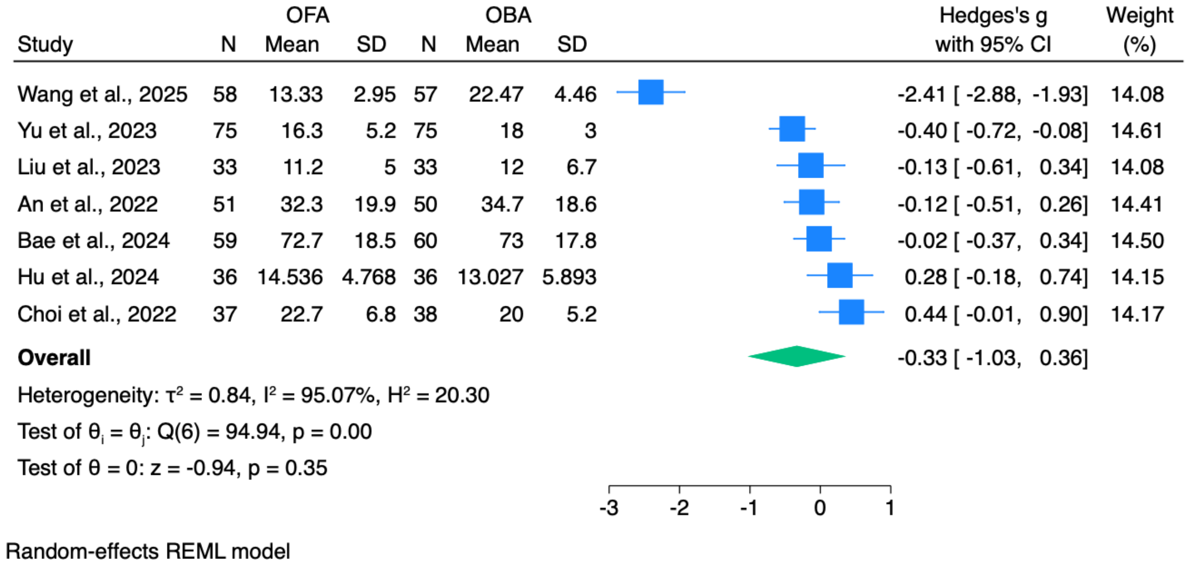

f) Forest plot comparing postoperative **time to flatus** for treatment-OFA vs. control-OBA.

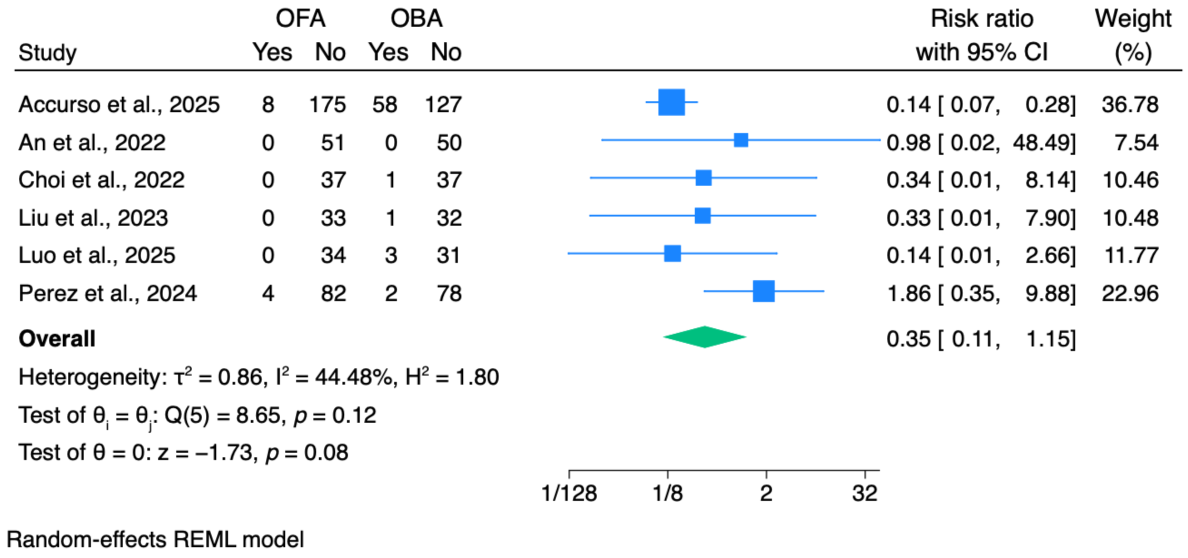

g) Forest plot showing pooled risk ratio for postoperative **pruritus** events for treatment-OFA vs. control-OBA.

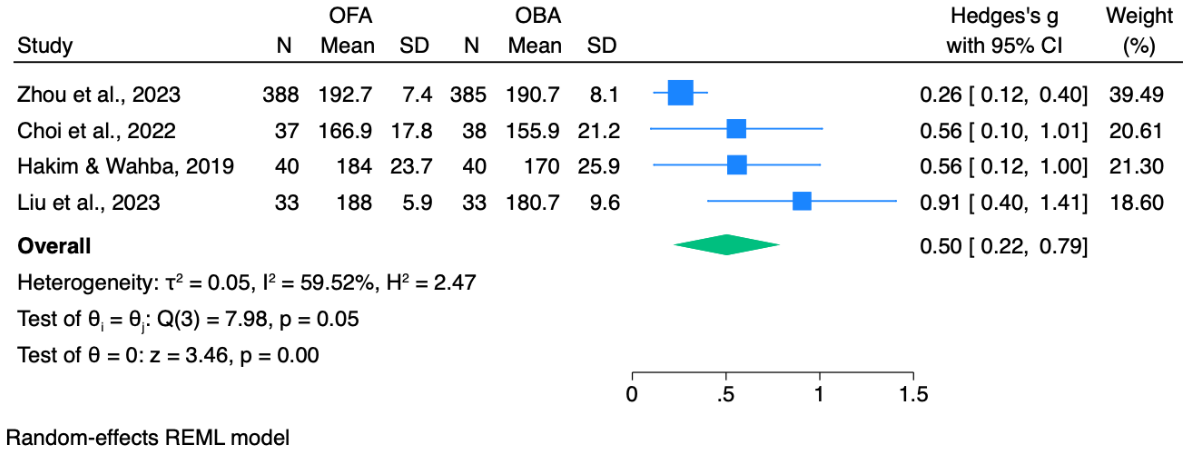

h) Forest plot comparing **Quality of Recovery-40** questionnaire for treatment-OFA vs. control-OBA.

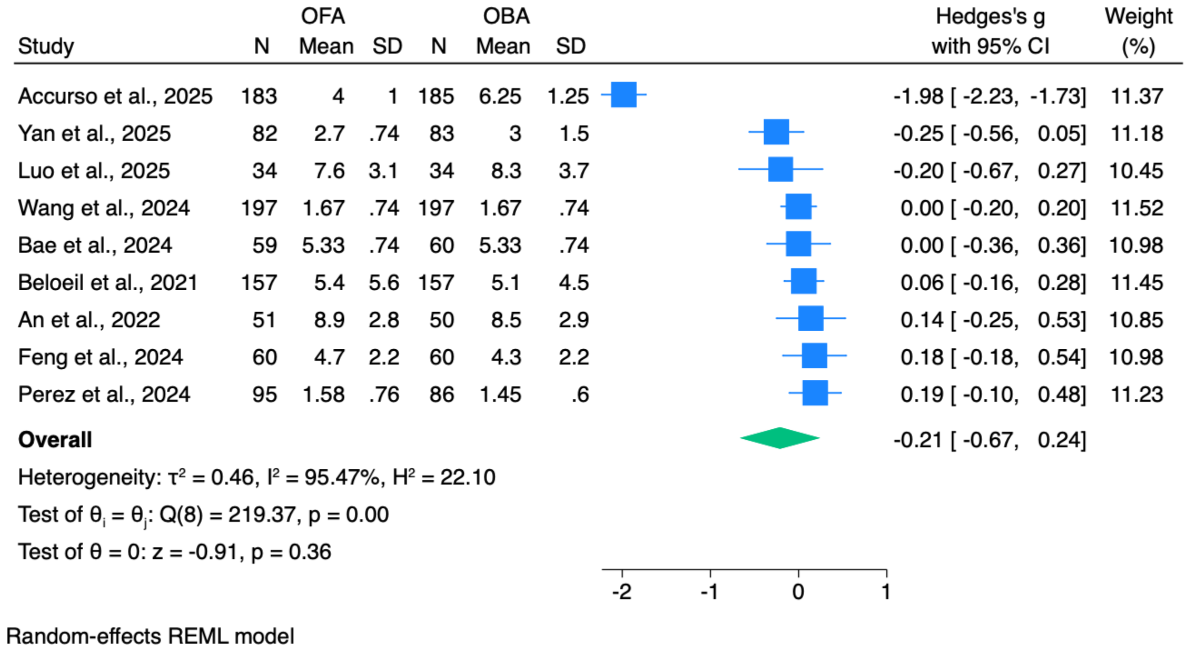

i) Forest plot comparing **hospital length of stay** for treatment-OFA vs. control-OBA.

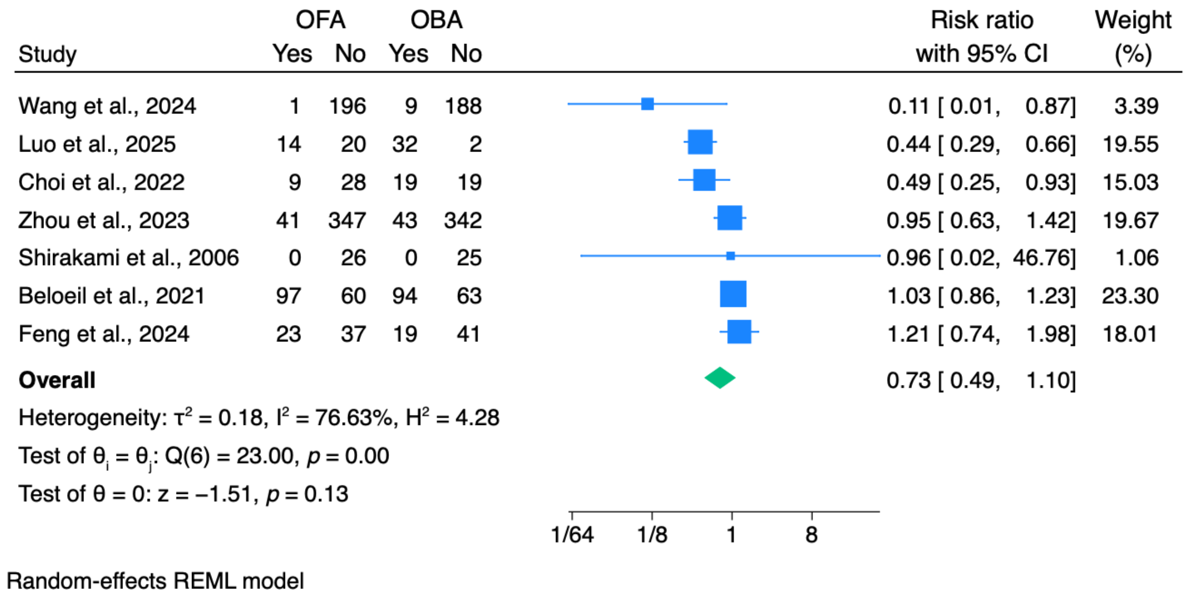

j) Forest plot showing pooled risk ratio for **intraoperative hypotension** for treatment-OFA vs. control-OBA.

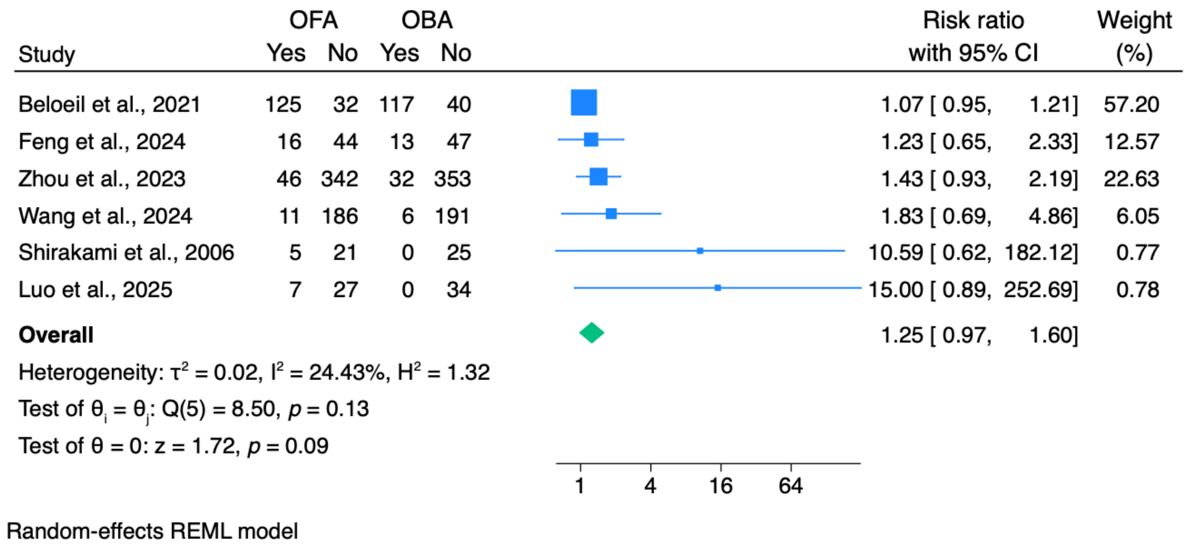

k) Forest plot showing pooled risk ratio for **intraoperative hypertension** for treatment-OFA vs. control-OBA.

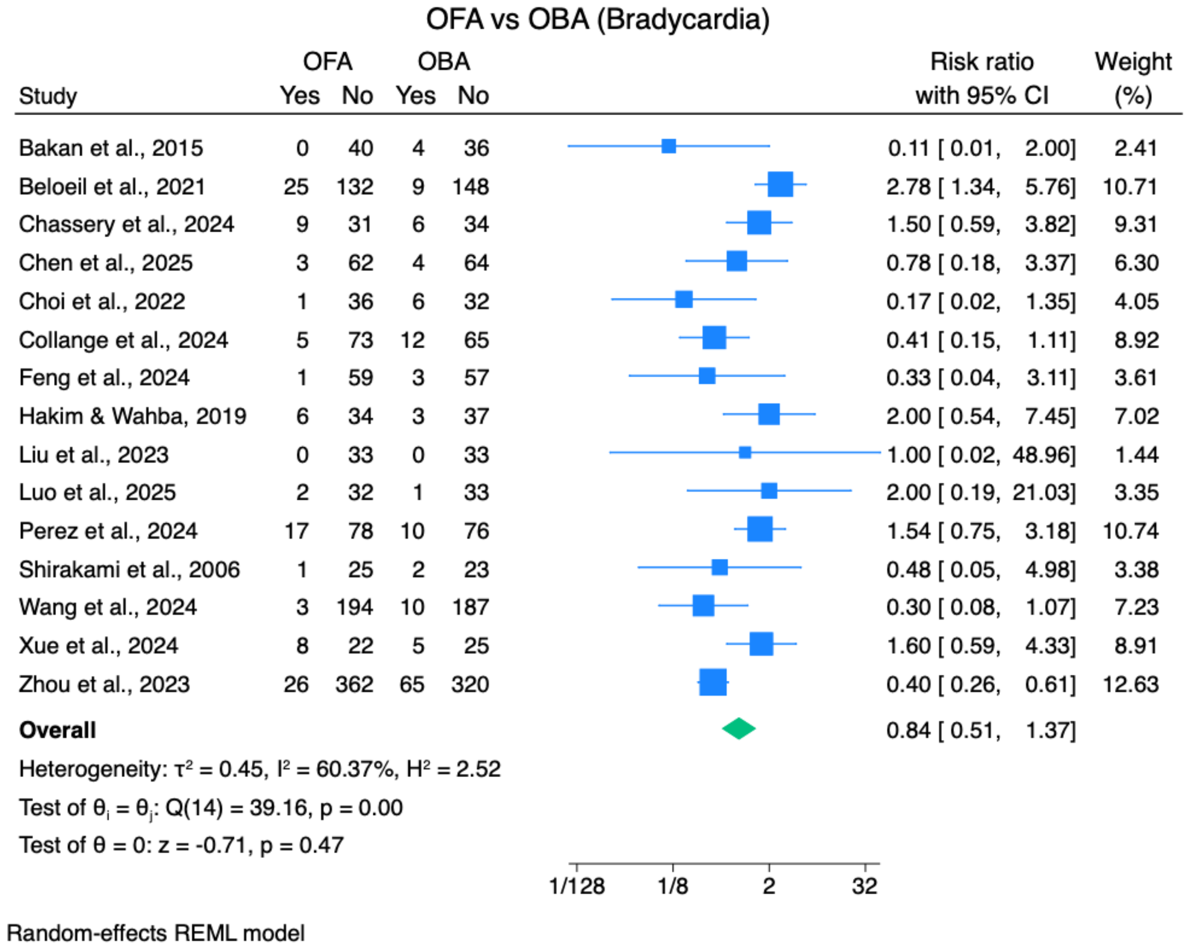

l) Forest plot showing pooled risk ratio for **intraoperative bradycardia** for treatment-OFA *versus* control-OBA.

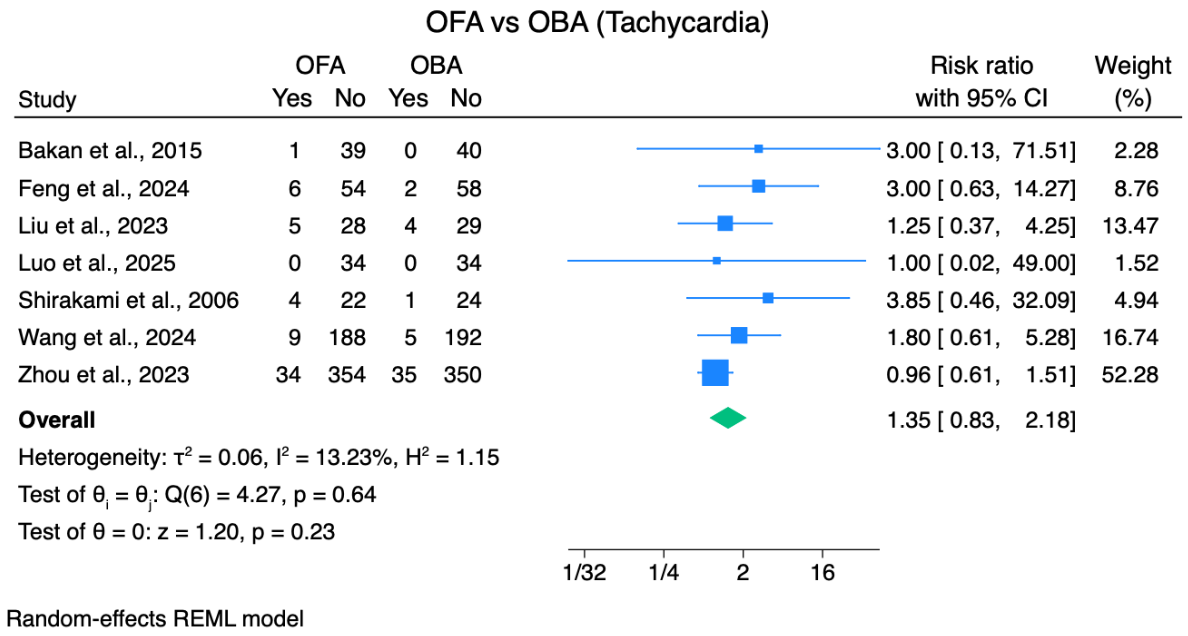

m) Forest plot showing pooled risk ratio for **intraoperative tachycardia** for treatment-OFA *versus* control-OBA.

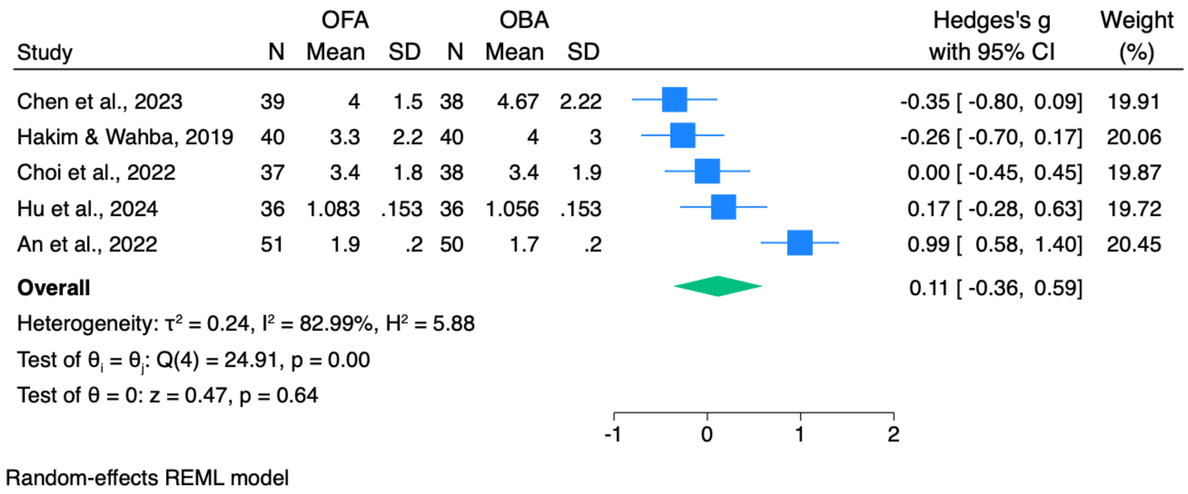

n) Forest plot showing pooled Hedges's g for **pelvic laparoscopic surgeries** between treatment-OFA *versus* control-OBA.

Pooled estimates of the standardized mean difference 95% CI are shown for the overall estimate of effect (*green diamond*). The standardized mean difference estimates for each study are represented as *blue squares*, and the *blue lines* represent traditional 95% CIs.

Abbreviations: CI – confidence interval; H^2 - ratio of total variability to sampling variability; I^2 - proportion of total variation due to heterogeneity; OBA - opioid-based anaesthesia; OFA - opioid-free anaesthesia; PONV – postoperative nausea and vomiting; Q - between-study heterogeneity test; QoR-40 - Quality of Recovery-40 questionnaire; REML - Restricted Maximum Likelihood; SD – standard deviation; τ^2 - estimated variance of true effect sizes between studies; τ - standard deviation of the true effects; z - test statistic assessing whether the overall pooled effect differs significantly from zero.
