# Supplementary material for: Role of Opioid-Free Anesthesia Versus Opioid-Based Anesthesia in Postoperative Pain and Opioid Consumption: A Systematic Review and Meta-Analysis
Source: J Clin Med. 2026 Jun 12;15(12):4560. doi: 10.3390/jcm15124560 (PMC13301896; doi:10.3390/jcm15124560)
Supplement: Supplementary file 1 [file jcm-15-04560-s001.zip › Supplementary File S8 - Funnel plot.pdf]

Supplementary File S8 – Publication bias assessment

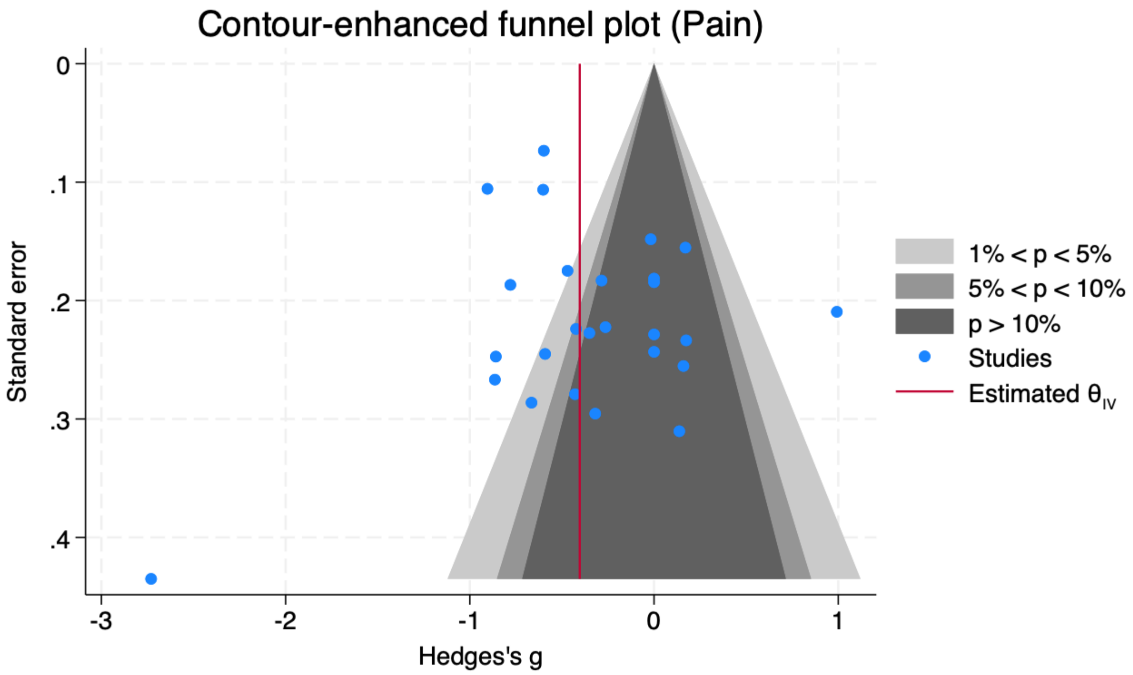

a) Contour-Enhanced Funnel Plot of Hedges' g for Pain Outcomes

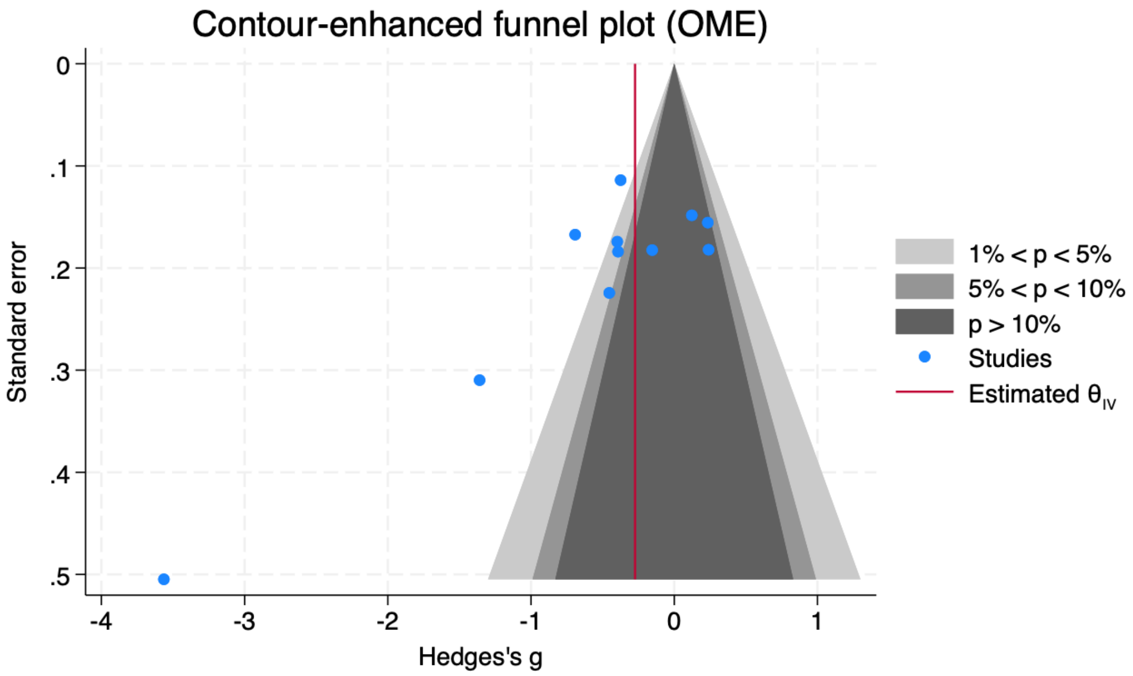

b) Contour-Enhanced Funnel Plot of Hedges' g for Oral Morphine Equivalent Outcomes

Note: Each blue point represents an individual study included in the meta-analysis. The x-axis shows the standardized mean difference or Hedges’ g, and the y-axis shows the corresponding standard error, with larger studies appearing toward the top of the plot. The red vertical line indicates the pooled effect estimate (θ_{IV}) from the inverse-variance model. Shaded regions represent contours of statistical significance based on two-sided p-values (light gray: 1% < p < 5%; medium gray: 5% < p < 10%; dark gray: p > 10%). Asymmetry in the distribution of studies may suggest the presence of small-study effects or publication bias.
